# The neuronal K^+^Cl^−^ co-transporter 2 (*Slc12a5*) modulates insulin secretion

**DOI:** 10.1038/s41598-017-01814-0

**Published:** 2017-05-11

**Authors:** Shams Kursan, Timothy S. McMillen, Pavani Beesetty, Eduardo Dias-Junior, Mohammed M. Almutairi, Abu A. Sajib, J. Ashot Kozak, Lydia Aguilar-Bryan, Mauricio Di Fulvio

**Affiliations:** 1 Department of Pharmacology and Toxicology, Wright State University, School of Medicine, Dayton, OH 45435 USA; 2 Pacific Northwest Diabetes Research Institute, Seattle, WA 98122 USA; 3 Department of Neuroscience, Cell Biology and Physiology, Wright State University, School of Medicine, Dayton, OH 45435 USA; 4 Department of Genetic Engineering and Biotechnology, University of Dhaka, Dhaka, Bangladesh

## Abstract

Intracellular chloride concentration ([Cl^−^]_i_) in pancreatic β-cells is kept above electrochemical equilibrium due to the predominant functional presence of Cl^−^ loaders such as the Na^+^K^+^2Cl^−^ co-transporter 1 (*Slc12a2*) over Cl^−^extruders of unidentified nature. Using molecular cloning, RT-PCR, Western blotting, immunolocalization and *in vitro* functional assays, we establish that the *“neuron-specific”* K^+^Cl^−^ co-transporter 2 (KCC2, *Slc12a5*) is expressed in several endocrine cells of the pancreatic islet, including glucagon secreting α-cells, but particularly in insulin-secreting β-cells, where we provide evidence for its role in the insulin secretory response. Three KCC2 splice variants were identified: the formerly described KCC2a and KCC2b along with a novel one lacking exon 25 (KCC2a-S25). This new variant is undetectable in brain or spinal cord, the only and most abundant known sources of KCC2. Inhibition of KCC2 activity in clonal MIN6 β-cells increases basal and glucose-stimulated insulin secretion and Ca^2+^ uptake in the presence of glibenclamide, an inhibitor of the ATP-dependent potassium (K_ATP_)-channels, thus suggesting a possible mechanism underlying KCC2-dependent insulin release. We propose that the long-time considered *“neuron-specific”* KCC2 co-transporter is expressed in pancreatic islet β-cells where it modulates Ca^2+^-dependent insulin secretion.

## Introduction

The pancreatic islet is remarkably sensitive to acute changes in plasma glucose levels. Glucose elevation of any magnitude triggers insulin secretion from β-cells to maintain blood glucose homeostasis. The initial or first phase of insulin secretion depends on cationic and anionic mechanisms. The K_ATP_-channel pathway underlies most of the cationic mechanisms and is the most thoroughly characterized so far^1^. The anionic mechanisms, independent of K_ATP_-channels, are linked to a much less characterized glucose-induced electrogenic Cl^−^ efflux^2^. Such efflux of Cl^−^ is possible because [Cl^−^]_i_ in β-cells is kept above that predicted by Nernstian equilibrium *i.e*., ~35 mM *vs*. ~10 mM^3–6^. In immature neurons^7^, neurons of the dorsal root ganglion^8, 9^ or adult chromaffin cells^10^, [Cl^−^]_i_ is also kept above thermodynamic equilibrium due to the predominant functional presence of the secondary active Cl^−^ loader *Slc12a2* (NKCC1), a bumetanide- (BTD)-sensitive Na^+^K^+^2Cl^−^ co-transporter, over the K^+^Cl^−^ co-transporters (KCCs), including KCC2 (*Slc12a5*), a constitutively active Cl^−^ extruder. In rat sensory neurons^9^ or bovine chromaffin cells^11^, the expression of NKCC1 is more abundant than that of KCC2. This is consistent with the observation that these cells actively accumulate Cl^−^ and exhibit [Cl^−^]_i_ above equilibrium (~35–40 mM)^10, 12^. In mature hippocampal^13^ or neocortical neurons^14^, for instance, the NKCC1/KCC2 mRNA ratio is ~1:2 due to developmental up-regulation of KCC2 expression^7, 14^. This is considered to be responsible for the generation and maintenance of [Cl^−^]_i_ below thermodynamic equilibrium (*e.g*., ~4 mM) in these mature neurons, thereby determining the well-defined inhibitory action of γ-aminobutyric acid (GABA) receptor type A (GABA_A_), a Cl^−^ channel gated by GABA, barbiturates or benzodiazepines^7, 14^. Accordingly, GABA_A_ activation in most mature neurons results in plasma membrane hyperpolarization due to Cl^−^ influx whereas in nociceptors it results in the electrogenic efflux of Cl^−^ and plasma membrane depolarization^8, 15^. This widely accepted model and established paradigm^14^ may be applied to any electrically excitable cell type expressing GABA_A_ and exhibiting [Cl^−^]_i_ above Nernstian equilibrium. Remarkably similar, mammalian insulin-secreting β-cells accumulate Cl^−^ in a BTD-sensitive manner^16, 17^, are depolarized by GABA^6, 18^ and upon opening of various Cl^−^ channels^19, 20^. These include the cystic fibrosis transmembrane conductance regulator CFTR^21, 22^ and Ca^2+^-dependent^4, 21, 23^ or volume-regulated Cl^−^ channels^24, 25^.

The predominant functional presence of NKCC1 relative to other Cl^−^ loaders expressed in rodent β-cells such as *Slc12a1* ^17, 26, 27^, some *Slc4a/26a* Cl^−^/HCO_3_^–^ exchangers^28, 29^ or any other potential Cl^−^ extruder of the *Slc12a* family^30^, partially explains the depolarizing driving force of Cl^−^ linked to insulin secretion^3, 19, 20, 31, 32^. Rat pancreatic islets express several Cl^−^ extruders including *Slc12a4* (KCC1), *Slc12a6* (KCC3) and *Slc12a7* (KCC4), however, these transporters appear to be enriched in glucagon-secreting α-cells. Indeed, the role of KCCs in cell volume regulation could not be demonstrated in dissociated rat β-cells subjected to hypotonic shock^30^, which is a classic maneuver to demonstrate KCC activity in many cell types^33^. The facts that KCC2 is a constitutively active Cl^−^ extruder refractory to hypotonic shock^34, 35^, and K^+^Cl^−^ co-transport activity is measurable in mouse pancreatic β-cells under basal conditions^36, 37^ raise the possibility that KCC2 is functionally present in β-cells. Recent data suggest that NKCC1 and KCC2 transcripts are co-expressed in human islets^38^, an observation strikingly similar to that of immature or sensory neurons^9^ or chromaffin cells^11^. In fact, human β-cells^6^, immature neurons^7^, nociceptors^39^ and adrenal medullary cells^11, 40^ all depolarize in response to GABA_A_ agonists, which matches with the demonstrated [Cl^−^]_i_ above thermodynamic equilibrium in these cells^5, 7, 10, 12^. Accordingly, acute inhibition of NKCC1 with the clinically relevant diuretics BTD or furosemide, inhibits GABA_A_-mediated plasma membrane depolarization of immature neurons^41^, nociceptors^39^, chromaffin cells^11^ and insulin secretion^5, 16, 17, 27, 31, 42^, respectively. Notably, these diuretics impair glucose tolerance in mice^27, 43–45^ and provoke intermittent hyperglycemia in patients treated with these compounds^46^.

The objective of the present work was to determine and characterize the expression patterns of *Slc12a5* gene products in the rodent/mammalian pancreatic islet and to determine if KCC2 plays a modulatory role in insulin secretion. We demonstrate that β-cells co-express three variants of KCC2 *i.e*., KCC2a, KCC2b and KCC2a-S25 (a novel KCC2a variant lacking exon 25), and pharmacological inhibition or stimulation of KCC2 in β-cells reciprocally modulate insulin secretion in response to glucose. In addition, inhibition of KCC2 increases insulin secretion by mechanisms involving a rise in cytosolic Ca^2+^ in a K_ATP_-channel independent-manner. Therefore, our results provide the first indication that KCC2 is expressed in the pancreatic β-cell and plays a role as a modulator of insulin secretion.

## Results

### Pancreatic β-cells express KCC2

**Figure 1:**
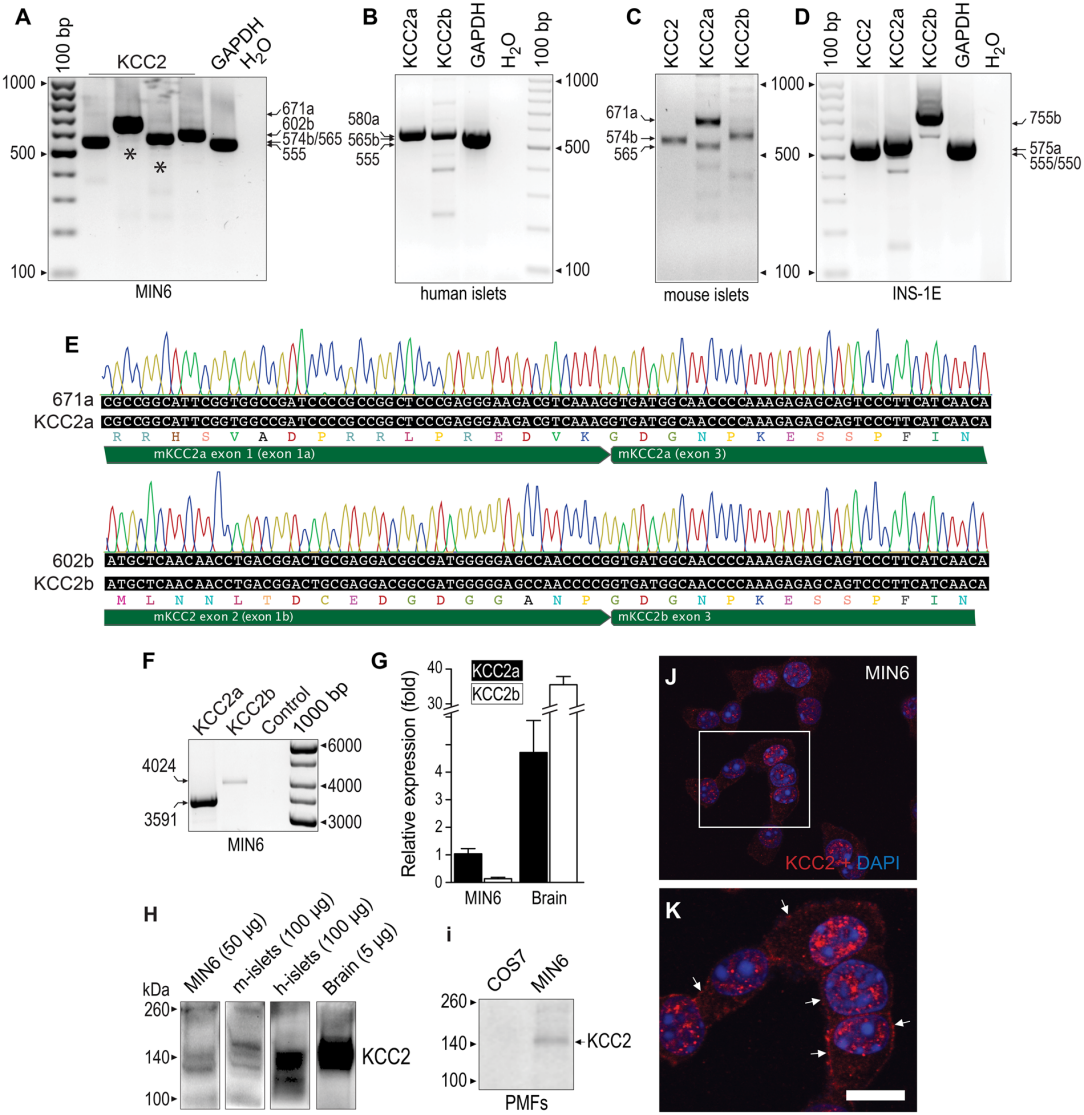
KCC2 is expressed in rodent β-cell lines and pancreatic islets. (**A**–**D**) Original ethidium bromide stained gels (inverted) showing KCC2 mRNA expression in the mouse β-cell line MIN6 (**A**), human (**B**) and mouse islets (**C**) and the rat INS1-E β-cell line (**D**). Shown are PCR products of expected sizes representing KCC2a and KCC2b. As the positive control of RT-PCRs, transcripts of GAPDH were amplified (555 bp). As the negative control, water was used instead of total cDNA. (**E**) Partial chromatograms obtained from representative DNA sequencing reactions using purified KCC2a-671 and KCC2b-602 PCR products from MIN6 (*black asterisks*). The DNA sequences obtained are 100% identical to the spliced version of KCC2a and KCC2b, respectively. (**F**) Representative original 1% agarose gel digital image cropped to include bands of relevant sizes. RT coupled to long-range PCR using total RNA from MIN6 and primer sets KCC2a-3591 or KCC2b-4024 (Supplementary Table 1). The PCR products of 3591 bp (KCC2a) and 4024 bp (KCC2b) were purified from this gel, directly cloned in pCR-Blunt II-TOPO vectors and fully sequenced in both directions. (**G**) MIN6 and mouse brain KCC2a and KCC2b mRNA quantification (*n* = 5). Results are expressed relative to MIN6 KCC2a. (**H**) Cropped digitised immunoblots of the indicated protein extracts (µg) obtained from MIN6, mouse (m) and human (h) islets and mouse brain. (**I**) Digitized immunoblot cropped to show KCC2 protein expression in purified plasma membranes of COS7 cells and MIN6 β-cells. (**J,K**) Confocal images of MIN6 β-cells immunolabeled using validated KCC2 antibodies. The square in (**J**) is shown at higher magnification in K to indicate with white arrows immunoreactive KCC2 towards the edges of the cells. Nuclei have been counterstained with DAPI. Scale bar represents 10 µm.

We used reverse transcription PCR (RT-PCR) to demonstrate KCC2 expression in MIN6, INS-1E β-cell lines and mouse and human islets. Figure 1A–D shows KCC2a and KCC2b expression as bands of expected size and identity (Fig. 1E). Moreover, full-length KCC2a and KCC2b were amplified, quantified (Fig. 1F–G), cloned and sequenced from MIN6 (Supplementary Figure 1). Next, immunoblotting with validated KCC2 antibodies^47^ was performed to demonstrate KCC2 protein expression (Fig. 1H) as bands of molecular weights (MW) from ~124 kDa to ~240 kDa, which are expected for KCC2 core/high-mannose (~124 kDa), hybrid-type (130–135 kDa), complex N-glycosylated (~150–160 kDa) and dimers (~240 kDa)^47, 48^. Notably, mature KCC2 (~150 kDa) was detected in crude plasma membrane extracts of MIN6 cells but not in those of COS7 cells, which lack endogenous KCC2 (Fig. 1i). Further, confocal images show that a small proportion of immunoreactive KCC2 localizes near or at the plasma membrane in MIN6 cells (Fig. 1J–K), partially co-localizes with cadherin, a plasma membrane marker, in the mouse islet whilst sharing a similar location pattern with NKCC1 and GLUT2 (Supplementary Figure 2). To define the relative contribution of KCC2 to the total KCC pool in MIN6, we determined and quantified KCC1, KCC3 and KCC4 expression levels (Supplementary Figure 3).

### β-cells express a novel splice variant of KCC2a

**Figure 2:**
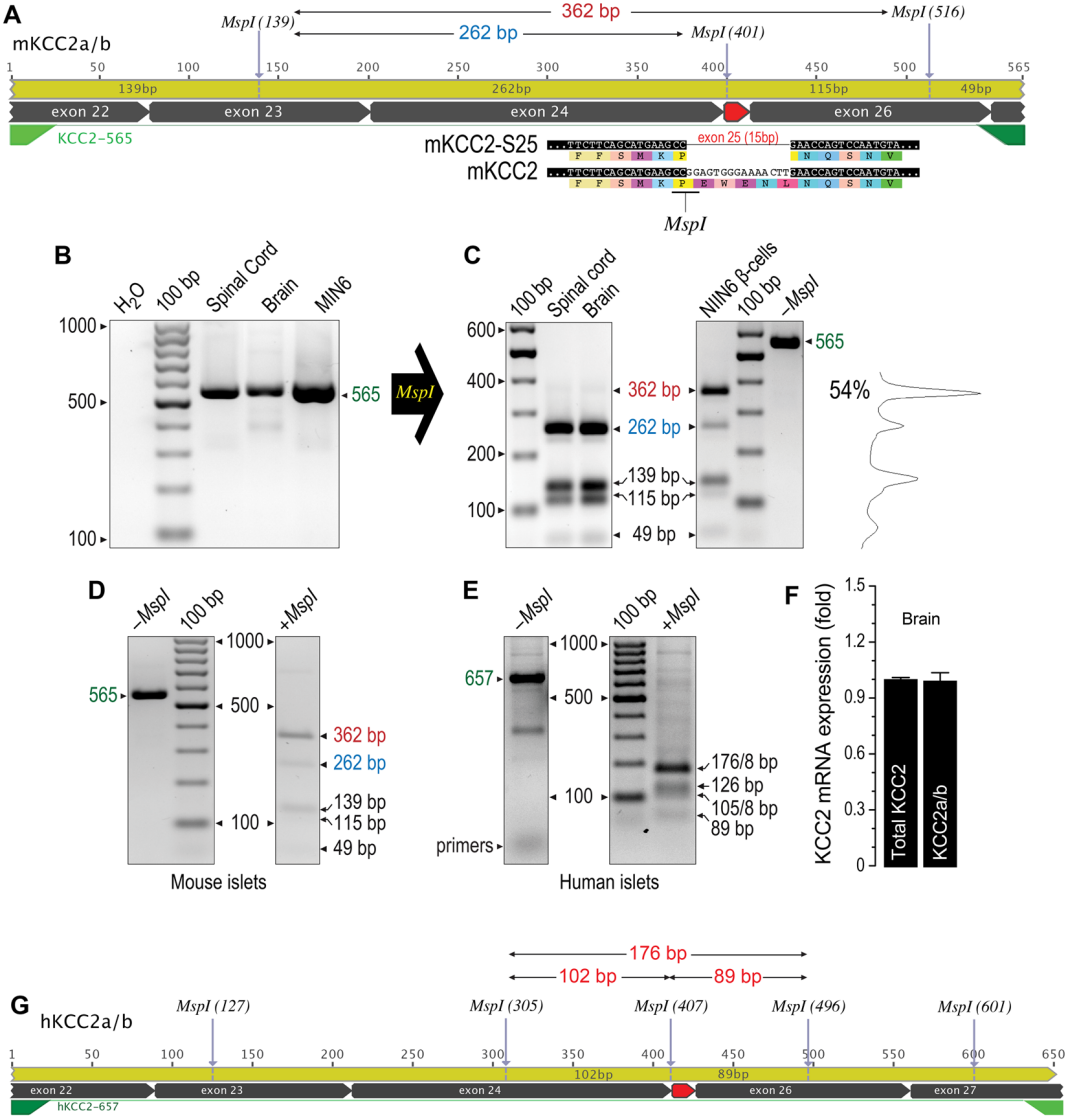
KCC2-S25 is expressed in MIN6 β-cells, human islets and mouse pancreas. (**A**) Representation of KCC2a/b amplicons obtained by using the KCC2-565 primer set. Indicated are the *MspI* restriction sites and the predicted length of the digestion products in bp. Exon 25 is highlighted in red. Its splicing eliminates an *MspI* site in the amplicon. (**B**) Ethidium bormide stained gel, inverted from its original gray-scale digital picture, showing RT-PCR products of expected size (565 bp) obtained by using the primer set KCC2-565 and total RNA from mouse spinal cord, brain and MIN6 β-cells. As negative control, water was used instead of total cDNA. (**C**) Representative ethidium bromide stained 2% agarose gel inverted from original where *MspI*-digested PCR products were separated. Also shown, representative densitometry analysis of the *MspI* banding pattern to estimate the relative contribution of KCC2-S25 (~54%) to the total KCC2 pool. (**D**) Representative ethidium bromide stained gel inverted from original showing an RT-PCR experiment performed using mouse islet RNA and the KCC2-565 primer set. Note the product of expected size and *MspI* digestion analysis of restriction fragments. (**E**) Representative ethidium bormide stained gel inverted from original showing RT-PCR experiment using total RNA from human islets and the KCC2-657 primer to obtain amplicons of expected size (657 bp) and *a posteriori MspI* digestion analysis. (**F**) Expression levels of total KCC2 in adult mouse brain using qPCR primers that do not distinguish among known KCC2 variants (total KCC2) or specific to exon 25 (KCC2a/b). (**G**) Representation of human KCC2a/b amplicons obtained using KCC2-657 primer set and predicted *MspI* restriction fragments for KCC2a/b-S25 (176 bp) and KCC2a/b (102 bp + 89 bp).

Three KCC2 splice variants were cloned from MIN6 and their sequences were deposited in NCBI *GenBank* (KJ535320, KJ535321 and KJ535322, Supplementary Figure 1C). KJ535322 matches mouse KCC2b (mKCC2b) and *RefSeq* NM_020333, whereas KJ535321 is similar to rat KCC2a (EF641113). Alignment of KJ535320 against mKCC2a demonstrated novel splicing involving nucleotides 3177–3191 and 3108–3122 in mKCC2a and mKCC2b, respectively, and corresponding to exon 25 of the mouse *Slc12a5* gene. This exon defines residues EWENL located in the predicted cytoplasmic C-terminus of KCC2a and KCC2b (Supplementary Figure 1A and C). This variant contributes to ~55–60% of the total KCC2 mRNA pool expressed in MIN6 (Fig. 2C and Supplementary Figure 1B). However, it was not detected in mouse adult brain or spinal cord (Fig. 2C and F).

To validate KJ535320 expression in β-cells, termed here as KCC2a-S25, the region encompassing exon 25 in KCC2 transcripts was PCR-amplified from MIN6, mouse brain, spinal cord, exocrine pancreas and human islets, and digested with *MspI*. Because an *MspI* site resides in the joint of exons 24–25 of *Slc12a5* transcripts, fragments of 362 bp and 262 bp demonstrate co-expression of KCC2-S25 and KCC2a/KCC2b, respectively (Fig. 2A). In human islets, *MspI* digestion of KCC2 amplicons produces bands of ~176 bp (Fig. 2E) whereas all KCC2 transcripts expressed in adrenal medullary cells lacked exon 25 (Supplementary Figure 4A–D). When taken together, these results suggest that KCC2-S25 represents an extra-neuronal KCC2 variant.

### KCC2 localizes to insulin-containing β-cells in mouse and human islets

**Figure 3:**
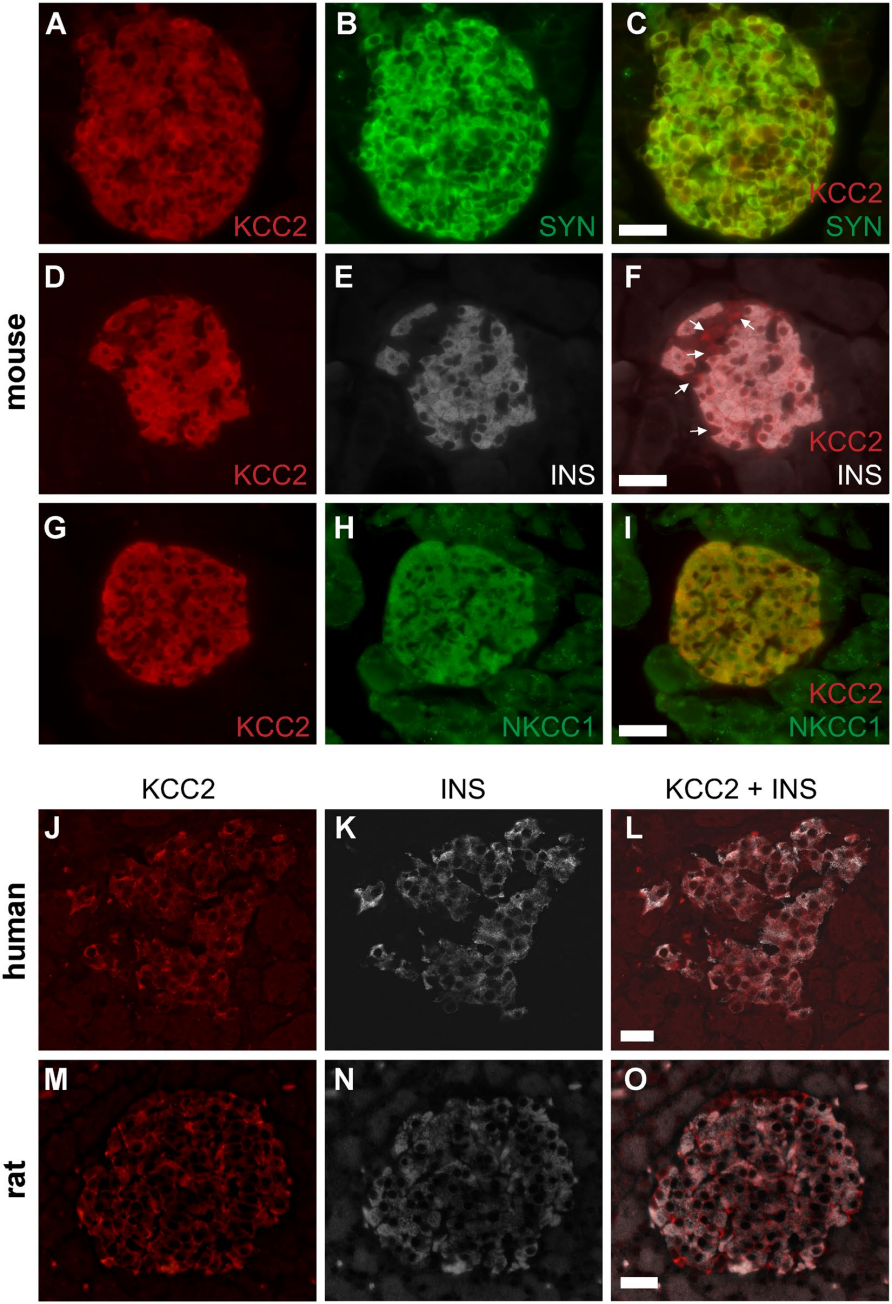
KCC2 locates to the endocrine islets of the mammalian pancreas. (**A–H**) Representative immunofluorescence microscopy images of mouse pancreas immunolabeled against endogenous KCC2 (**A**,**D** and **G**), synaptophysin (SYN, B), insulin (INS, E) or NKCC1 (**H**), using Cy3-, AF488- or DyLight405-labeled secondary antibodies [red (KCC2), green (SYN and NKCC1) and white (INS), respectively]. Also shown are superimposed pictures of KCC2- and SYN-, INS- and NKCC1-labeled images to visualize co-localisation [red + green = yellow (C and I) and red + white = pink (F)]. Arrowheads in F indicate KCC2-positive/INS-negative islet cells. (**J–O**) Shown are confocal immunofluorescence microscopy images of human (**J**–**L**) and rat (**M**–**O**) pancreas tissues immunolabeled using KCC2 (red) and INS (white) antibodies. Scale bars represent 50 µm.

Immunofluorescence microscopy was employed to complement the results on KCC2 expression from β-cell lines and show localisation in the pancreatic islets. To identify them, pancreas sections were stained with synaptophysin (SYN, pan-endocrine marker). Figures 3A–C show KCC2 staining specifically in endocrine cells of the pancreas. To define these endocrine cell types, KCC2 and insulin were co-immunolabeled. Figure 3D–F shows that KCC2 co-localizes with insulin (INS), whereas a substantially lower level of KCC2 was found in insulin-negative cells (*arrows*, Fig. 3F). These cells are identified in their majority as glucagon-positive α-cells of the mouse islet (Supplementary Figure 5). Together, these results demonstrate that, within the islet, insulin-secreting β-cells contain most of the immunoreactive KCC2. Because NKCC1 is expressed in isolated β-cells of the rat islet^49^ and rodent β-cell lines or islets^26, 27^ we verified that KCC2 and NKCC1 also co-localize in the mouse islet. To this end we used NKCC1 antibodies validated against NKCC1^KO^ tissues^50^. As shown in Fig. 3G–I, KCC2 and NKCC1 co-localise in the mouse islet β-cells and both co-transporters co-localise with cadherin (Supplementary Figure 3). To further validate these results, KCC2 was simultaneously labeled in 5 µm thick tissues sections of mouse brain, spinal cord, kidney, salivary gland and pancreas positioned within the same chamber (Supplementary Figure 6A–F). Moreover, two KCC2 monoclonal antibodies were used to label the co-transporter in mouse pancreas and in COS7 and MIN6 cells (Supplementary Figure 6G–O). We extended these results to human and rat pancreas sections (Fig. 3J–O).

### KCC2 modulates insulin secretion in response to glucose

**Figure 4:**
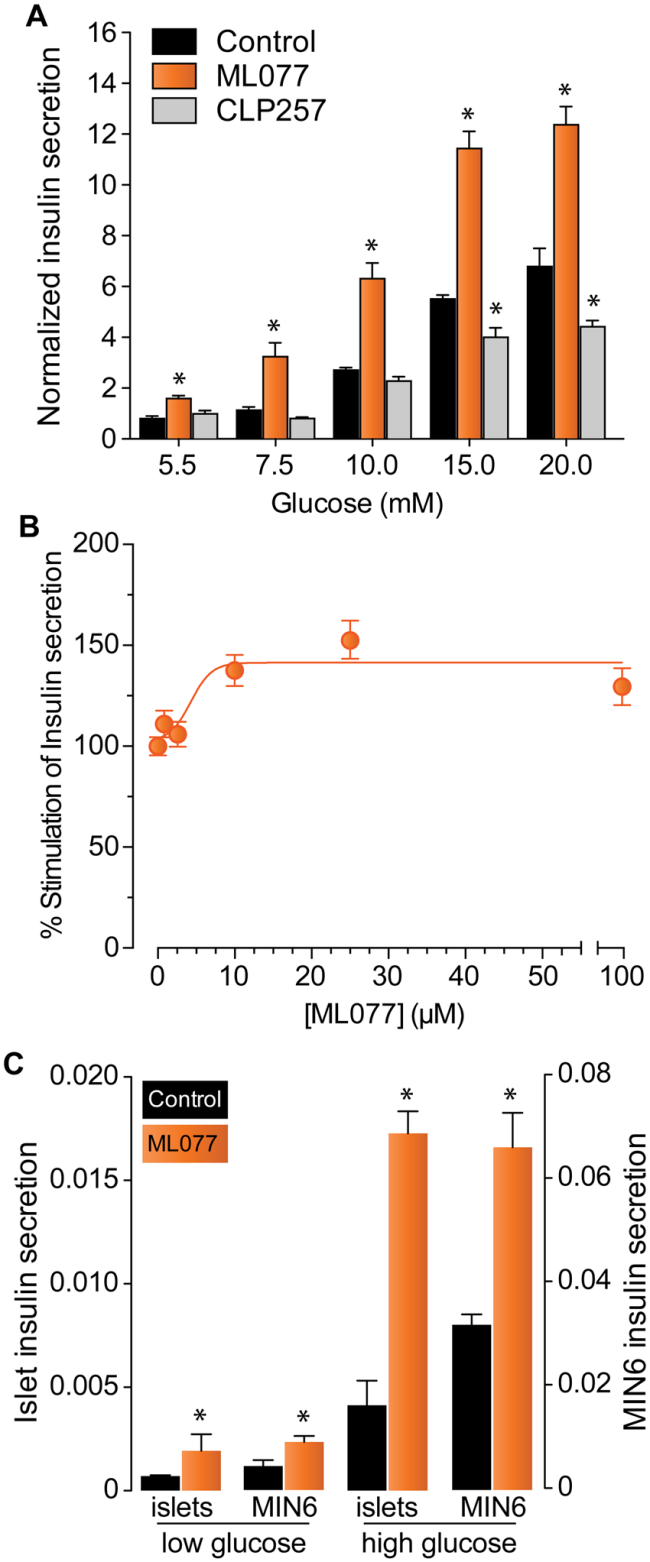
Insulin secretion in MIN6 and islets is modulated by KCC2. (**A**) Insulin secretion in MIN6 β-cells under basal (5.5 mM glucose) or stimulated conditions (7.5–20 mM glucose), either in the presence of ML077 (25 µM) or CLP257 (100 µM). Results are expressed as percentage of total insulin content and normalized to Control 5.5 mM ± SEM (*n* = 3, *p < 0.05). (**B**) Dose-response curve of the basal secretory response (5.5 mM glucose) of MIN6 β-cells as a function of increasing concentrations of ML077 (1–100 µM). Shown are the results of 2 different experiments performed in triplicate. (**C**) Insulin secretion of mouse islets and MIN6 incubated in low or high glucose (3.3–16.6 mM for islets or 5.5–15 mM for MIN6, respectively). Results of insulin secretion are expressed relative to total insulin content (*n* = 3, *p < 0.05 *vs*. glucose alone).

NKCC-dependent decrease in β-cell total Cl^−^ content correlates with impaired insulin secretion^17^. Since KCC2 is a constitutively active Cl^−^ extruder, its pharmacologic modulation is therefore expected to alter [Cl^−^]_i_ and the secretory response. As shown in Fig. 4A, treating MIN6 cells with the KCC2 inhibitor ML077^51, 52^ at maximally effective concentrations (25 µM, Fig. 4B) significantly increases insulin secretion in response to 5.5 mM glucose, which is a physiologically normal and a non-insulinotropic concentration. The same effect is observed at higher concentrations of the sugar. Notably, the insulinotropic effect of ML077 in basal glucose was also observed with VU0240551, a less selective KCC2 inhibitor^53^ (Supplementary Figure 7A). However, CLP257 moderately inhibited insulin secretion only at high glucose concentrations (≥15 mM). Remarkably, neither ML077 nor CLP257 significantly changed the calculated EC_50_, *i.e*., the concentration of glucose giving half-maximal responses (~10 mM). Yet, ML077 and CLP257 significantly increased and decreased, respectively, the maximum insulin response to glucose. Therefore, ML077 and CLP257 increase and decrease, respectively, the efficacy of glucose to elicit insulin secretion. To further extend and confirm these results, we next tested the effect of ML077 on basal and glucose stimulated insulin secretion (GSIS) using mouse islets. As shown in Fig. 4C, mouse islets responded similarly to low (3.3 mM) and high (16.6 mM) glucose.

### KCC2-dependent insulin secretion does not require K_ATP_-channels: the role of Ca^2+^

**Figure 5:**
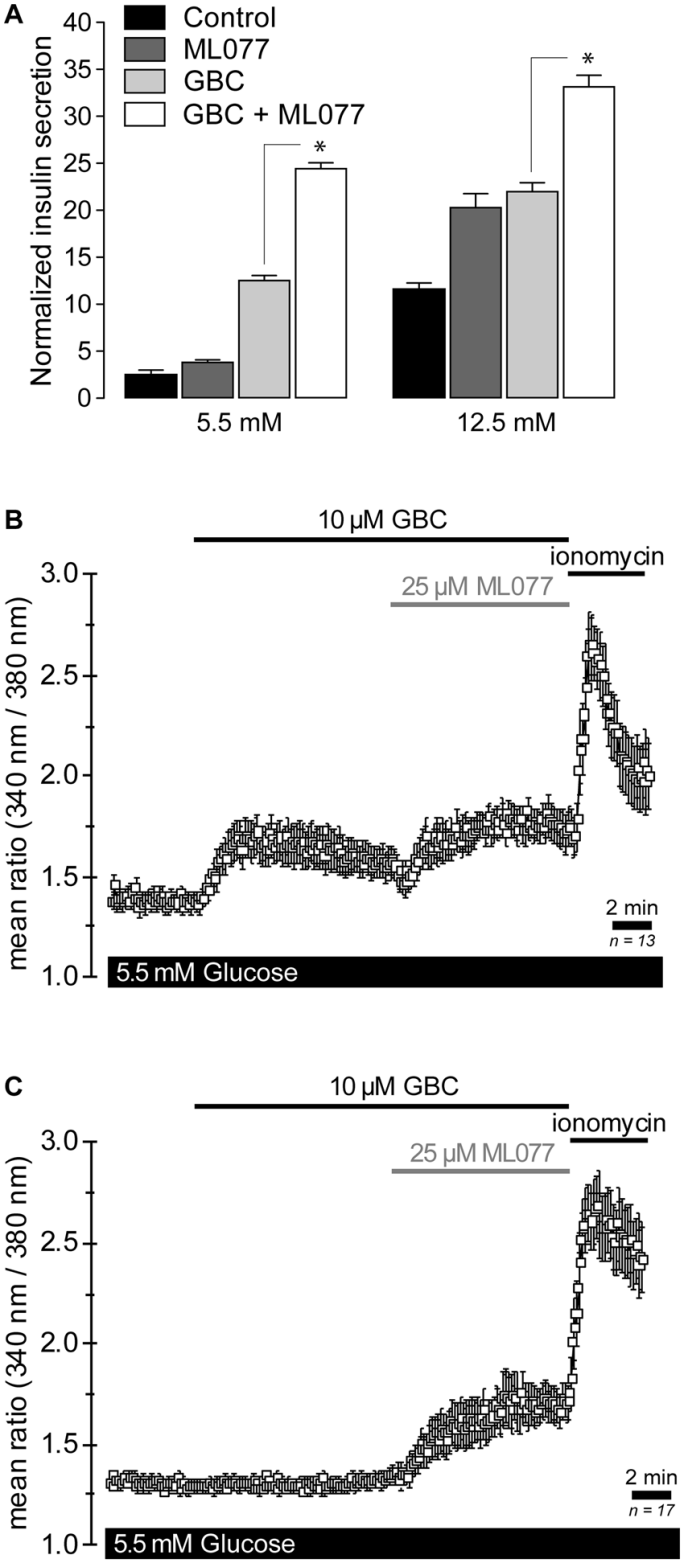
ML077 increases insulin secretion and glibenclamide-induced Ca^2+^ elevation in MIN6 β-cells. (**A**) Insulin secretion in MIN6 β-cells under low and high glucose conditions either in the presence of ML077 (25 µM, dark gray bars), glibenclamide (GBC 10 µM, light gray bars) or both (empty bars). Results are expressed as percentage of total insulin content (*n* = 3, *p < 0.05). (**B**) Single-cell Ca^2+^ imaging in Fura2-loaded MIN6 β-cells (*n* = 13) under basal secretory conditions (5.5 mM glucose). Addition of 10 µM GBC evoked a rapid cytosolic Ca^2+^ elevation, which was increased by 25 µM ML077. (**C**) Experiment identical to A in a group of cells that did not exhibit Ca^2+^ elevation in response to 10 µM GBC. Addition of 25 µM ML077 in the presence of 10 µM GBC evoked Ca^2+^ increases. In B and C, ionomycin (10 µM) was applied at the end of the experiment to estimate maximum Ca^2+^ responses. Each symbol represents a mean 340 nm/380 nm fluorescence ratio from 13 and 17 cells (**B** and **C**) ± SEM.

The inactivation of K_ATP_-channels and the resulting plasma membrane depolarization to a threshold that activates Ca^2+^ entry through voltage-dependent Ca^2+^ channels play a key role in the first phase of insulin secretion^54^. Therefore, we tested the effect of ML077 on the basal secretory response of MIN6 β-cells in the presence of glibenclamide (GBC, 10 µM), a K_ATP_-channel inhibitor. As shown in Fig. 5A and Supplementary Figure 7B, GBC increased insulin secretion in response to 5.5 mM and 12.5 mM glucose, whereas nifedipine (10 µM), an inhibitor of L-type Ca^2+^ channels prevented those effects, as expected. However, ML077 alone or in combination with GBC significantly increased basal and GSIS (Fig. 5A). These data suggest that the positive effect of ML077 on insulin secretion observed under basal or stimulated conditions may not require activated K_ATP_-channels. To further examine KCC2-modulated basal insulin secretion, we measured changes in cytosolic Ca^2+^ in response to GBC (10 µM) and ML077 (25 µM) in MIN6 cells loaded with Fura-2. Application of GBC resulted in a significant elevation in cytosolic Ca^2+^ in the majority of cells, as expected (Fig. 5B). Further, addition of ML077 in the presence of GBC significantly increased intracellular Ca^2+^ levels, which lasted for several minutes. Because a small proportion of MIN6 cells do not respond to GBC^55^, we examined the effect of ML077 in cells where GBC alone did not evoke a Ca^2+^ increase. As shown in Fig. 5C, ML077 robustly increased Ca^2+^ uptake in GBC-insensitive MIN6 cells uncovering a KCC2-sensitive Ca^2+^ influx pathway in these cells.

## Discussion

KCC2a, KCC2b and a novel KCC2a-S25 variant were cloned from pancreatic β-cells (Fig. 1 and Supplementary Figure 1). KCC2a and KCC2b differ at the N-terminus, but their basal transport activities are equivalent^56^ and both contribute to normal Cl^−^ homeostasis in neurons^14^. The presence of unique N-terminal phosphorylation sites in KCC2a, which are absent in KCC2b, raises the possibility of differential regulatory mechanisms^56^. This concept may have functional significance in β-cells, particularly when considering that KCC2a and KCC2a-S25 make up ~90–95% of the total KCC2 pool in β-cells (Fig. 1G and Supplementary Figure 1B), whereas KCC2b accounts for ~90–95% of the total KCC2 pool in the adult mouse brain^56^. Clearly, KCC2a-S25 cloned from β-cells direct the translation of KCC2 proteins when transfected into cell lines (Supplementary Figures 4F,G) suggesting that KCC2a-S25 mRNAs are translationally active. Further, it is unlikely that KCC2a-S25 is the result of cloning artifacts. Indeed, by taking advantage of a unique *MspI* site in the junction of exons 24–25, we demonstrated that KCC2 transcripts lacking exon 25 are endogenously expressed in β-cells (Fig. 2A and F) and in adrenal medullary cells (Fig. 2B–E and Supplementary Figures 4A–D). Further, no evidence of KCC2-S25 mRNA expression in the adult brain or spinal cord could be found (Fig. 2C and F).

KCC2 expression was also demonstrated in mouse islets, MIN6 and αTC6 cell lines’ immunoblots using validated antibodies (Fig. 1H, Supplementary Figures 5K and 6G–I). Irrespective of the KCC2 antibody used, the co-transporter was identified in SYN-labeled endocrine cells in the islet, but not in the exocrine portion of the mouse, rat or human pancreas (Fig. 3 and Supplementary Figures 6E,F and 6M–O), and in the medulla of the rat adrenal gland (Supplementary Figure 4A,B). Therefore, the low likelihood that a diverse panel of antibodies could detect a similar non-specific expression pattern and label anatomically defined tissue regions suggests specificity for the KCC2 protein. In fact, the KCC2 antibody used in our experiments *i.e*., 07–432 have been recently validated using knockout tissues^57^. Unlike mature neurons, which express abundant, but not exclusively plasma membrane localised KCC2^58, 59^, a substantial proportion of β-cell KCC2 was found in intracellular compartments, like NKCC1^9, 50^, NKCC2^17, 26, 27^, KCC3^60^ and KCC4^61^ in other cell types. This is not surprising; ~90–95% of the total NKCC immunoreactivity localise in intracellular compartments, when using cell lines^62^ and a minute amount could be demonstrated in the plasma membrane when using mouse islets cells (Supplementary Figures 2A,D and G). Indeed, a small fraction of total immunoreactive KCC2 localises towards the MIN6 cells edge (Fig. 1J–K), the plasma membrane of endocrine/islet mouse cells (Supplementary Figures 2B,E and H) and αTC6 cells (Supplementary Figure 5J). Further favoring the proposal that complex N-glycosylated KCC2 is the one enriched at the plasma membrane^48^, KCC2 was detected as a single band of ~150 kDa in purified plasma membrane fractions of MIN6 β-cells, but not in those of COS7 cells (Fig. 1I).

Although the functional properties of KCC2a-S25 remain to be determined, the fact that mKCC4 cloned from several sources (*GenBank* accessions: AK166215, AK149750, BC059242, AK143535, AK131129, BC141107, AF087436) lacks exon 27 (homologous to exon 25 in *Slc12a5*), adds an extra layer of complexity to our current understanding of the molecular physiology of KCC2, particularly when considering that KCC2a-S25 accounts for ~55–60% of total KCC2 transcripts in β-cells (Supplementary Figure 1B). KCC2a-S25 lacks five C-terminal residues located immediately after the so-called “ISO-domain”, which is responsible for the high basal activity of the transporter^35^ whereas KCC4, which lack ISO domains, is an inactive cotransporter under normal isotonic conditions^35, 63, 64^. However, unlike KCC2, KCC4 is robustly activated by hypotonicity^64^. Hence, it is tempting to speculate that KCC2a-S25 and KCC4 may share functional properties. Interestingly, the total KCC2 transcript level in MIN6 β-cells is ~10–15 times more abundant than that of KCC4 (Supplementary Figure 3B). Thus, when taking into consideration that total KCC activity in β-cells is measurable^36^ but not involved in regulatory volume decrease in response to hypotonic challenge^30^, these data suggest that KCC2a and/or KCC2a-S25 may take part in a portion of net Cl^−^ transport in β-cells.

At the functional level, inhibition of NKCC (a Cl^−^ loader) with BTD impairs insulin secretion at all concentrations of glucose tested^5, 16, 31, 42^. However, stimulation of KCC2 (a Cl^−^ extruder) with CLP257 decreased insulin secretion only in response to high glucose (Fig. 4A), whereas inhibition of KCC2 with ML077 increased basal and stimulated insulin secretion in MIN6 cells and mouse islets (Fig. 4C). Although small decreases in [Cl^−^]_i_ are known to alter the oscillatory Ca^2+^ signaling in murine β-cells^5, 23^, our experiments cannot exclude the possibility that changes in [Cl^−^]_i_ may also affect insulin secretion by slightly changing the intragranular pH^65^ or granule exocytosis, as previously suggested^20^. When coupled to active V-type H^+^-ATPase-dependent H^+^ uptake, vesicular Cl^−^/H^+^ transporters are expected to electrically neutralize and acidify the organelle’s lumen^66^. Interestingly, only high glucose (15 mM) caused an acute and sustained Ca^2+^-independent drop in vesicular pH^67^ and CLP257 decreases insulin secretion in response to ≥15 mM glucose (Fig. 4A). It is worth noting that β-cell KCC2 partially co-localized with SYN (Fig. 3A–C), a marker of endosomes synaptic-like macrovesicles (SLMVs) where the 2Cl^−^/H^+^ exchanger Clc-3 was found^68^. However, while SLMVs do not store insulin, they appear to concentrate at least GABA and glycine^69^ raising the possibility that KCC2 may modulate secretion indirectly by participating in SLMV maturation/release of these Cl^−^ channel activators. Clearly, further experiments are needed to determine the potential impact of KCC2 in β-cell granule biology as well as in the physiology and interplay of endocrine cells of the islet were KCC2 is also expressed (see Supplementary Figure 5).

Notably, ML077-induced insulin secretion in response to 5.5 mM and 12.5 mM glucose also occurred in the presence of GBC, but was inhibited by nifedipine (Fig. 5A and Supplementary Figure 7B) as expected, suggesting that KCC2 modulates the secretory response independently of K_ATP_-channels. Together, these data support the recent findings demonstrating that the electrogenic exit of Cl^−^ from β-cells is required to broaden Ca^2+^-dependent membrane potential oscillations and to increase the density of action potentials needed to sustain insulin secretion in response to glucose^5, 23^, phenomena that occur when K_ATP_-channels are fully closed^23^. Indeed, according to the current paradigms and the laws of thermodynamics, the electrogenic driving force of Cl^−^ is expected to increase when [Cl^−^]_i_ increases in β-cells in response to KCC2 inhibition. In fact, such Cl^−^ channel-mediated depolarizing exit of Cl^−^ has been recorded, even in the absence of functional K_ATP_-channels^70^. Further, the Cl^−^ driving force and the electrogenic Cl^−^ conductance positively correlate with the magnitude of Ca^2+^ entry in neurons^71^.

Consistent with these data, single-cell Ca^2+^ measurement experiments exhibited that ML077 increases GBC-induced Ca^2+^ elevations in MIN6 β-cells (Fig. 5B,C). Ca^2+^ elevations observed in response to GBC are believed to represent an amplified opening of voltage-dependent L-type Ca^2+^ channels in response to plasma membrane depolarisation^1^ brought about by K_ATP_-channel inhibition, and in effect mimic the situation during glucose-induced K_ATP_-channel closure. Although the detailed mechanisms underlying the stimulation of Ca^2+^ uptake (and insulin secretion) either directly or indirectly through potential changes in [Cl^−^]_i_ remain to be dissected, our results thus far are compatible with several non-exclusive interpretations: *i*) increased recruitment of active Ca^2+^ channels in response to ML077 (Fig. 5B), *ii*) shifting of the plateau membrane potential to more depolarized levels (expected if [Cl^−^]_i_ increases in response to KCC2 inhibition, as demonstrated in other cells^52^) and the consequent opening of additional Ca^2+^ channels further increasing Ca^2+^ influx^72^, *iii*) increasing Cl^−^-channel mediated efflux and action potential rate/density, as recently demonstrated^23^ and/or *iv*) Cl^−^, independently of its conductance, directly shifting the gating of L-type Ca^2+^ currents to the left, as demonstrated for other anions^73^. Notably, in β-cells that did not initially respond to GBC with a rise in cytosolic Ca^2+^ ML077 evoked a significant Ca^2+^ increase (Fig. 5C) indicating that ML077 *per se* is able to promote Ca^2+^ entry and suggesting that in low/basal glucose conditions (*e.g*., 5.5 mM glucose) K_ATP_-channels counteract most of the depolarizing Cl^−^ conductance in β-cells.

Whilst treatment of MIN6 cells with ML077, considered a highly selective and specific inhibitor of KCC2^52^, correlated with increased basal insulin secretion (Fig. 4), drug-mediated inhibition/stimulation of unknown targets does remain possible. Nevertheless, it is also plausible that the modulation of insulin secretion by ML077 reflects KCC2-dependent changes in [Cl^−^]_i_. For instance, in immature neurons with measured [Cl^−^]_i_ of ~35mM^74^ and equilibrium potential for Cl^−^ (E_Cl_) of ~−37 mV, a 5 mM *decrease* in [Cl^−^]_i_ profoundly impacted the ability of GABA to promote Ca^2+^ fluxes in these cells^75–77^. Similarly, [Cl^−^]_i_ in mouse β-cells is ~34 mM^5^ and have a predicted E_Cl_ of ~−37 mV (if cultured with external [Cl^−^]_o_ of 142.6 mM, as given by the KRBH buffer in our experiments). Therefore, taking into consideration the electrophysiological similitudes between immature neurons and β-cells in terms of [Cl^−^]_i_ and E_Cl_ it is reasonable to expect that small changes in [Cl^−^]_i_ in β-cells will produce similar electrophysiological results as those described in immature neurons.

When taken together, these findings are consistent with a small albeit significantly stimulated insulin secretion in response to non-insulinotropic glucose in the presence of ML077 (and other KCC2 inhibitors, see Supplementary Figure 7A) and with the fact that inhibition of KCC2 increases the maximal insulin response to glucose (Fig. 4A). Therefore, the observed increase in insulin secretion in response to ML077 is supported by increased Ca^2+^ influx under conditions where [Cl^−^]_i_ is increased, as expected when the constitutively active KCC2 is inhibited. In addition, ML077 neither increased cytosolic Ca^2+^ nor insulin secretion in the presence of nifedipine, a dihydropyridine blocker of L-type Ca^2+^ channels (Supplementary Figures 7B,C). These results are indeed expected when considering the proposal that Ca^2+^-independent pathways contribute minimally to GSIS^78^. However, they also suggest that the modulatory effect that KCC2 inhibition has on insulin secretion does not involve potential secretory mechanisms, which are not dependent on Ca^2+^ entry such as hypotonicity-induced cell swelling^79^ or increased cAMP^80^. Although it has been reported that β-cell membrane potential does not change in response to 1.1 mM, 3.3 mM or 5.5 mM glucose^81^, a detailed analysis as that performed recently^23^ will be needed to elucidate the mechanisms whereby KCC2 inhibition promotes Ca^2+^ entry. Taken together, these data provide evidence supporting the conclusion that the constitutively active Cl^−^ extruder KCC2 is expressed in β-cells and participates in the modulation of the secretory response.

## Methods

### Materials and immunochemicals

Platinum *Pfx* thermostable DNA polymerase, RNaseOUT, SuperScript-III reverse transcriptase, random hexamers, Lipofectamine2000 and tissue culture media were from Invitrogen (Carlsbad, CA); dNTPs and molecular biology grade chemicals were from Affimetrix (Cleveland, OH); custom PCR/mutagenesis primers were from Integrated DNA Technologies (IDT, Coralville, IA). Restriction/cloning enzymes were from New England Biolabs (Ipswich, MA). High-fidelity *Pfu* Ultra II Fusion HS DNA polymerase and the QuickChange-II XL site-directed mutagenesis kit were from Agilent (Santa Clara, CA). Tissue culture supplements and general chemicals were from Sigma-Aldrich Co. (St. Louis, MO). Culture media low in glucose (5.5 mM) was from Hyclone-GE (Logan, UT). Microscopy materials were from EMS (Hatfield, PA) and Thermo Fisher Sci. (Waltham, MA). Collagenase type IV from *Clostridium histolyticum* (≥160 U/mg) was from Worthington (Lakewood, NJ). To assess KCC2 function, we used KCC2 inhibitors VU0240551 (Tocris, Minneapolis, MN) and VU0255011/ML077 (Aobious Inc., Gloucester, MA)^51, 52^ and the agonist CLP257^82^, originally a kind gift of Dr. Yves De Koninck (Université Laval, Québec) and later on commercially procured from Tocris (Minneapolis, MN). Glibenclamide and nifedipine were from R&D (Minneapolis, MN). β-actin (JLA20), KCC2 (07–432) and insulin (274A-15) antibodies were from DSHB (University of Iowa), EMD Millipore (Billerica, MA) and Cell Marque Corp. (Rocklin, CA), respectively. Antibody against glucagon (K79bB10) and synaptophysin (ab130436) were from Abcam (Cambridge, MA). Monoclonal KCC2 antibodies (clones N1/12 and N1/66) were from Neuromab (UC Davis, CA). Monoclonal antibodies against pan-Cadherin (clone CH-19, MA1079) were from BosterBio (Pleasanton, CA). Rabbit antibodies against rat glucose transporter-2 (GLUT2, GT21-A) or GAPDH (G9545) were from Alpha Diagnostic (San Antonio, TX) and Sigma (Saint Louis, MO), respectively. Secondary Cy3-, AlexaFluor (AF488)- and DyLight405-conjugated antibodies were from Jackson (West Grove, PA).

### β-Cell culture and insulin secretion

Mouse MIN6 and rat INS-1E β-cells were kind gifts of Jun-Ichi Miyazaki (Medical School, Osaka University, Japan)^83^ and Pierre Maechler (University Medical Centre, Geneva, Switzerland)^84^, respectively. The mouse glucagon-secreting α-cell line αTC6 (αTC1 clone 6) and COS7 cells were from ATCC (Manassas, VA). Cells were cultured and grown in regular DMEM supplemented with 50 µM β-mercaptoethanol, 15% heat-inactivated fetal bovine serum (FBS), 100 IU/ml penicillin and 100 µg/ml of streptomycin. Cells were maintained inside an incubator at 37 °C in a humidified environment having 5% CO_2_ until ~80–90% confluence was attained. Media were changed every 2–3 days. β-cells were cultured in low glucose (5.5 mM) media 24 h before experiments. COS7 cells were cultured as indicated^50^. Basal or glucose-stimulated insulin secretion (GSIS) was determined in MIN6 β-cells two hours after incubation in the presence of low (5.5 mM) or high glucose concentrations (7.5–20 mM) by ELISA (EZRMI-13K, EMD Millipore, Billerica, MA) in accordance with the manufacturer’s directions. The following procedure was followed: MIN6 β-cells were cultured in 12-well plates in low-glucose DMEM overnight. Then, cells were washed twice with sterile glucose-free Krebs-Ringer HEPES buffer (KRBH: in mM, 135 NaCl, 3.6 KCl, 5 NaHCO_3_, 0.5 Na_2_HPO_4_, 0.5 MgCl_2_, 1.5 CaCl_2_, 10 HEPES pH 7.4 and 0.1% BSA, 290–300 mOsm/Kg H_2_O) and incubated in KRBH + 5.5 mM glucose for 30 min at 37 °C. After a final wash in this medium, cells were incubated 2 h in KBRH supplemented with 5.5–20 mM glucose alone or in combination with drugs. Released insulin was determined in cleared supernatants after an overnight incubation at −20 °C. Total insulin content of attached cells was determined in acidified ethanol (75% ethanol/1.5% HCl) extracts. Insulin secretion was expressed as the ratio of secreted insulin into the media to the sum of secreted and intracellular content.

### Islet isolation for gene expression and secretory response

Experimental protocols involving purification of mouse islets were performed at the University of Washington (Seattle, WA), the approving institution, Cell Function Analysis Core of the Diabetes Research Center (DRC). All methods were carried out in accordance with relevant guidelines and regulations. C57BL/6 J male and female wild-type (WT) mice (6–10 week old) were sacrificed and pancreatic islets isolated for gene expression analysis essentially as described^17^. Purified islets were immediately used for protein/RNA extraction. Islets for secretory studies were isolated from 8–10 week old C57BL/6 J mice by the DRC Cell Function Analysis Core as described^85^. Islets were cultured overnight in RPMI1640 and hand picked into individual wells of 12-well plates (with mesh inserts) containing KRBH (in mM: 118.5 NaCl, 2.5 CaCl_2_, 1.2 KH_2_PO_4_, 4.7 KCl, 25 NaHCO_3_, 1.2 MgSO_4_, 10 HEPES and 0.1% BSA pH 7.4, 290–300 mOsm/Kg H_2_O) plus 3.3 mM glucose. The mesh inserts containing islets were transferred to new wells containing KRBH + 3.3 mM glucose and incubated at 37 °C (5% CO_2_) for 30 minutes. This wash step was repeated once more. The islets were then transferred into their respective experimental wells containing KRBH + 3.3 mM glucose plus vehicle (DMSO) or the indicated concentrations of KCC2 inhibitors for 2 h at 37 °C (5% CO_2_). Islets were transferred into new wells containing KRBH + 16.7 mM glucose plus vehicle or KCC2 inhibitors, incubated 2 h at 37 °C (5% CO_2_) and transferred to new wells containing acidified ethanol. The KRBH from experimental wells was frozen at −20 °C until analysis. Insulin secreted into the media or contained in cells was estimated by using ELISA.

### RT-PCR, long-range PCR and cloning

Total RNA for RT-PCR was obtained from freshly isolated mouse islets, α- or β-cell lines using the RNeasy mini kit (Qiagen, Valencia, CA) and quantified by using NanoDrop ND-1000 Spectrophotometer (Thermo Sci/NanoDrop Wilmington, DE). Total RNA extracted from normal human islets was kindly provided by Dr. Patrick MacDonald (University of Alberta, Edmonton, Canada). Mouse tissues RNA for control experiments were from Zyagen (San Diego, CA). RT-PCR was performed as described^26^. The sets of primers used to amplify KCC2 and control transcripts are indicated in Supplementary Table 1. RT-PCR amplicons were sequenced (Beckman Genomics, Beverly, MA) and aligned/assembled *in silico* against rat/mouse/human sequences of reference (RefSeq) using Geneious Suite R7-9 (Biomatters Ltd., New Zealand). The open-reading frames (ORFs) plus un-translated regions (UTRs) of KCC2 transcripts were obtained by using long-range PCR as follows: 2 µg of total RNA were reverse transcribed as indicated above and amplified by PCR using 0.2 U of *Pfu*, 400 µM dNTPs, 1 mM MgCl_2_ and 1 µM each transcript-specific primer (Supplementary Table 1). The thermal conditions for PCR were: 1 min denaturation at 95 °C, 40 cycles (0.3 min at 95 °C, 2 min at 59 °C, 2 min at 72 °C) and a final extension at 72 °C (10 min). The long-range PCR products were purified, directly cloned (pCRII-Blunt-TOPO system) and sequenced. RT-PCR products or their digestion products were resolved on 1–2% agarose gels stained with ethidium bromide. Gels were visualized in a UV transiluminator and directly photographed by using the ChemiDoc™ MP Imaging System with Image Lab™ Software (Bio-Rad, Berkeley, CA). The gray-scale digital images obtained were inverted for clarity and cropped to exclude gel edges or non-relevant lanes.

### Real-time quantitative RT-PCR

cDNA was synthesized from 1 μg total RNA using QuantiTect Reverse Transcription Kit (Qiagen, Valencia, CA). Reverse transcribed KCC1, KCC2, KCC3, KCC4 and L32 (housekeeping gene) mRNAs were quantified by PCR using the StepOnePlus™ Real-Time PCR System, the ABI Fast SYBR Green Master Mix (Applied Biosystems, Foster City, CA) and specific primers (Supplementary Table 2). PCR reactions were performed in triplicate and the data analyzed with the ΔΔC_*t*_ method as described^86^. After normalization to internal control L32, the results were expressed as fold change relative to KCC2a or total KCC2.

### Mutagenesis, tagging and expression

KCC2 cDNAs inserted in pCR-Blunt II-TOPO were mutagenized to introduce a unique *SfiI* site at position –5 (relative to start codon A^1^TG) necessary for further sub-cloning/tagging. Mutagenesis was performed using the following sense/antisense primers: 5′-GTG CGA TCC CGC GGC CCC GGA GGC C **AT G**AG CCG C-3′ and 5′-GCG GCT CAT GGC CTC CGG GGC CGC GGG ATC GCA C-3′ (the *SfiI* site is underlined, first codon in bold). Mutagenized inserts were cloned into *SfiI*/*XhoI* sites of the pCMV-*myc* mammalian expression vector (Clontech, Mountain View, CA). After sequencing confirmation, pCMV-*myc* plasmids were transfected into COS7 or MIN6 β-cells to verify expression.

### Western blotting

Proteins from isolated islets and cell lines were obtained as described^50^. Protein extracts from human islets were kindly provided by Dr. Patrick MacDonald. Briefly, up to 100 µg of total or membrane proteins purified by using the Subcellular Protein Fractionation kit for cultured cells were loaded onto pre-casted 4–20% Tris-HEPES protein gels (Thermo Scientific-Pierce), run under denaturing conditions and electro-transferred onto PDVF membranes at 4 °C. Transferred membranes were cut into two pieces using the 70 kDa protein marker as a reference to allow independent but simultaneous immunolabeling of sample proteins in the top and bottom portions of the membrane. After blocking, the PVDF membranes were incubated with KCC2 and β-actin antibodies, respectively, and subsequently with proper secondary HRP-antibodies. We used ChemiDoc™ MP Imaging System with Image Lab™ Software (Bio-Rad, Berkeley, CA) to detect antigen-antibody reactions directly on PVDF membranes. High-resolution digital images were taken and lanes of interest cropped to exclude irrelevant lanes. PVDF membranes involving subcellular fractions (membranes and cytoplasmic extracts) were simultaneously labeled against KCC2 and GAPDH as a control and developed using a rabbit HRP-conjugated secondary antibody.

### Immunofluorescence microscopy

MIN6 β-cells or αTC6 α-cells cultured on glass coverslips or tissue sections from mouse, human pancreas (Cell Marque Corp) and pancreas sections from streptozotocin (STZ)-induced diabetic mice [a kind gift of Dr. Khalid Elased (WSU)] were immunolabeled as described^17, 50^. Labeled slides were immediately viewed using an Olympus Epi Fluorescence Spot microscope equipped with RT color camera. Digital images were obtained using a Diagnostics Instrument Spot 6 digital camera (Spot Imaging Solutions, Sterling Heights, MI). High-resolution confocal images were taken by using the FV1000 Confocal Microscope (Olympus, PA, USA). Co-localisation Finder and RGB Profiler tools in *ImageJ* (NIH)^87^ were used to estimate co-localisation of NKCC1, KCC2, GLUT2/insulin with cadherin, a plasma membrane marker. When DyLight405-conjugated antibodies were used to visualize insulin-positive β-cells, images were taken in gray-scale instead of blue color to increase contrast against red- and green-stained antigens.

### Fluorescence Ca^2+^ imaging

MIN6 cells were plated on acid washed glass coverslips and cultured in regular media for 2–3 days. One day before the experiment, the media was replaced to low glucose DMEM (5.5 mM glucose). On the day of the experiments, glass coverslips with attached cells were moved to the 35 mm glass-bottom imaging chamber with solution volume of ~1 ml. The cells were loaded with Fura-2AM ratiometric calcium indicator dye (Thermo Fisher Scientific, Waltham, MA) for ~90 min at 37 °C in the recording solution. For complete Fura-2AM de-esterification, the dye-containing solution was replaced with fresh recording solution and cells were incubated for an additional 30 min at 37 °C. The recording solutions were composed of Krebs-Ringer HEPES buffer of identical composition to that used for basal secretion experiments in MIN6 and supplemented with 5.5 mM glucose. The hypoglycemic agent, glibenclamide (GBC, 10 µM) was used to inhibit K_ATP_-channels and increase intracellular Ca^2+^ levels necessary for insulin secretion. At the end of each experiment ionomycin (10 µM, EMD Millipore, Billerica, MA) was used to estimate maximum Ca^2+^ responses. Solutions in the imaging chamber were exchanged with a syringe-driven perfusion system. Lambda 10B shutter and filter wheel (Sutter Instrument Company, Novato, CA) were used to illuminate individual cells in the imaging field at 340 nm and 380 nm wavelengths every 5 seconds. The light source was a 175 W Xe lamp (QED Medical, Lexington, KY). Pixelfly CCD camera (PCO Imaging, Kelheim, Germany) and InCyt Im2 software (Intracellular Imaging, Cincinnati, OH) was used to capture and analyze images. Light intensities emitted from individual cells were measured at 535 nm and the ratios plotted as a function of time using Origin software v8.6 and 2016 (OriginLab, Northampton, MA).

### Statistical analysis

Analysis of multiple group differences was performed using two-way analysis of variance (ANOVA) followed by Student-Newman-Keuls’ test. A *p* value less than 0.05 was used as the criteria for statistical significance.

## Electronic supplementary material

Supplementary Data
