# Supplementary material for: The neuronal K+Cl− co-transporter 2 (Slc12a5) modulates insulin secretion
Source: Sci Rep. 2017 May 11;7:1732. doi: 10.1038/s41598-017-01814-0 (PMC5431760; doi:10.1038/s41598-017-01814-0)
Supplement: Supplementary file 1 — Supplementary Data [file 41598_2017_1814_MOESM1_ESM.pdf]

Supplementary Data

The neuronal K^+Cl^- co-transporter 2 (*Slc12a5*) modulates insulin secretion

Shams Kursan[1], Timothy S. McMillen[2], Pavani Beesetty[3], Eduardo Dias-Junior[1], Mohammed M. Almutairi[1], Abu A. Sajib[4], J. Ashot Kozak[3], Lydia Aguilar-Bryan[2] and Mauricio Di Fulvio[1]*

[1]Department of Pharmacology and Toxicology, Wright State University, School of Medicine, Dayton, OH 45435; [2]Pacific Northwest Diabetes Research Institute, Seattle, WA 98122; [3]Department of Neuroscience, Cell Biology and Physiology, Wright State University, School of Medicine, Dayton, OH 45435; [4]Department of Genetic Engineering and Biotechnology, University of Dhaka, Bangladesh.

*Corresponding author: Mauricio Di Fulvio, Ph.D., Department of Pharmacology and Toxicology, Wright State University, School of Medicine, 3640 Colonel Glenn Hwy, 216 HSB, Dayton OH, 45435. Phone: (937) 775-5250, fax: (937) 775-7221. Mauricio.DiFulvio@wright.edu

Content

Table 1: The primer-sets used in RT-PCR experiments.

Table 2: The primer-sets used in quantitative RT-PCR.

Figure 1: Predicted primary structure of β-cell mKCC2a, mKCC2a-S25 and mKCC2b proteins.

Figure 2: Localisation of NKCC1, KCC2 and GLUT2 in the plasma membrane of islet cells.

Figure 3: KCC1, KCC3 and KCC4 mRNAs expression in MIN6 β-cells.

Figure 4: Expression of mKCC2-S25 in medullary adrenal cells.

Figure 5: KCC2 expression in glucagon-positive α-cells.

Figure 7: Validation of KCC2 antibodies.

Figure 7: Effects of VU0240511 and ML077 in the secretory response of MIN6 and the role of voltage-activated L-type Ca^{2+} channels.

Full size blots and gels: Shown are original blots and gels that were used to construct cropped Figures 1F, 1H and 1i in the main text.

Supplementary Table 1: The primer-sets used in RT-PCR experiments are named after the target transcript (KCC1-4, GAPDH) or variants (KCC2a-b, KCC3a-d), followed by numbers indicating amplicon sizes in base pairs (bp). Sense and antisense primer sequences encompass transcript positions, which are numbered relative to start codon A^1TG. Primers were designed according to mouse (m), rat (r) or human (h) cDNAs and/or *RefSeq* nucleotide sequences as indicated.

Primer set	Sense	Antisense	Position	GeneBank	Species
mKCC1-440	GTGACATCTCTGCCTATACCTATG	GACTTCAAGGAATTCCATGTAGTTC	2747-3186	NM_009195	mouse
mKCC1-529	CATGTTCTTTCTGATGTGTTACCTC	GACTTTCTCAATGTCCATCATGTTC	1746-2274		
mKCC1-531	GGCTCAGTTGGGTGGACTAC	ATTCTGGTCCCAAAGAGCGG	65-589		
mKCC2a-671	GCAGGTTCACGGTCACCTC	CAGGATCTCGATCGTGCCAA	7-678	XM_006499943	mouse
mKCC2b-574	CCAAAGAAAACCCGCCAGTG	TACACACCCATGAAGGTGCC	-263-311	NM_020303	mouse
mKCC2b-602	GCTCAACAACCTGACGGACT	TCTCGATCGTGCCAAGGATG	2-604		
mKCC2-314	GTCTTCAGTGATATGACCTCCTAC	CAGGTAGAGAAGAAAGAGCCTATG	1173-1487		
mKCC2-337	GTCATCGTCATAGGCTCTTTCTTC	GAGTCCAGTGGTAATAGCGAAATC	1455-1792		
mKCC2-345	GAAACAATGTCACAGAGATCCAGG	GAACAACAGAGCTGATGTAGACAG	1009-1354		
mKCC2-408	GATCATCAGACATGGAGGAACTTC	CATCTGTTTGAGGATCTGAGATCG	2349-2757		
mKCC2-565	GAGAAGACATTAGTAATGGAGCAAC	CCTCCAAGAATTCCATGTAGTTTTC	2709-3724		
mKCC2-569	GAGGTCATCGTGAATAAATCTCGG	TCTCCTGTACACTATGTATAGCGC	3168-3737		
mKCC2-598	CTATTACCACTGGACTCTCTCCTT	GAAGTTCCTCCATGTCTGATGATC	1775-2373		
mKCC2-693	GGAGTCCTTCTGTATGGTCTTCAT	GAACTCCACAGGTTCTCTTTGATG	389-1082		
mKCC2-813	GAAGTAGAGAGCATGAAGAAGCAG	CCTGGATCTCTGTGACATTGTTTC	220-1033	XM_006499943	mouse
mKCC2a-1s	CTGCGGCCACTTGTGCGATCC		-37		
mKCC2b-1s	CAAAGAAAACCCGCCAGTGGCTCA		-262	NM_020303	mouse
mKCC2-1a		TGTAAACGGTGACAGCGGCG	3438		
mKCC2-2a		CCAGGGCATGGGCAACTGGG	3463		
mKCC2-3a		GCAGGCAGCGGGAAGGAAAG	3486		
mKCC2-4a		CTCTCCACGCCTCCTCGCCA	3607		
mKCC2-5a		GGGGACATTAAAAATACACGCGATGTCTCC	3762		
rKCC2a-575	CAGGTTCACGGTCACCTCGCTG	GAGACCTGGAAATCATGTAGTAGGAGCCAC	8-583	EF641113	rat
rKCC2b-755	CATGCTCAACAACCTGACGGACTG	CGTACTTGACGCCCACAAAGACTAC	-1-754	NM_134363	rat
hKCC2a-502	GGTTCACGGTCACCTCGCTG	ATGGCCGTGAGCATCGTACA	10-512	NM_001134771	human
hKCC2a-580	GGGGGAAGACGTCAAAGGTGAT	AGCCAGCAGGATTTCGATGG	104-684		
hKCC2b-565	ATGCTAAACAACCTGACGGAC	TAGTGCCCAGGTAGAAGCAGAG	1-565	NM_020708	human
hKCC2b-597	CAACCCGGGTGATGGCAACC	AAGATGGCCATGGCTGGGAA	44-641		
hKCC2-657	GCTTACACCTATGAGAAGACGTTG	TTCTCAGGAGTAGATGGTGATGAC	2697-3354		
mKCC3-589	CCTACTGAAGCTGGATGAAGATTTAC	CTTCATCTGGATACTGTTGTCTTCTAG	2130-2718	NM_133648	mouse
mKCC3a-401	AGCAGAAGTAAAAGCCCGGA	CCCAGGTAAGACGCAGGAAG	78-478		
mKCC3b-695	GTGGGACACTCGGCAGATAA	CTGACTCGGGTCCTCCGTAA	-413-282	NM_133649	mouse
mKCC3c-675	CCCCAGGATGTTACGGAGGATG	GGCTGTACCATAGACGCGCA	253-927	XM_006498545	mouse
mKCC4-571	CATTTATCTTTCCTGCATAGTGCTG	GTACTCGATGTACTTGTAGATGCA	1392-1962	NM_011390	mouse
mKCC4-580	CTGCATCTACAAGTACATCGAGTA	CTCTTGGTTTTGTGGGAATAAGTC	1938-2517		
mKCC4-663	ATCAATAATGTGGAGGTGGAAAGAG	GACATATTTGACACCAACAAAGACC	157-819		
mKCC4-691	GAACATCTTGGGTGTTATCCTTTTC	GTTGTTCTGTGCAAAGTACTCATC	390-1080		
GAPDH-555	GTGAAGGTCGGAGTCAACGGATTT	CACAGTCTTCTGGGTGGCAGTGAT	9-564	NM_002046	human

Supplementary Table 2: The primer-sets used in quantitative RT-PCR are labeled according to the target mRNA followed by amplicon sizes in base pairs (bp). The positions encompassing both primers are relative to start codon A[1]TG. Primers were designed according to *RefSeq* nucleotide sequences of reference.

Primer set	Sense	Antisense	Position	GeneBank	Species
KCC1-129	ACCAAAACTGAGCGGGATC	CCACGTCATCTGGATCTTGTC	2824-2951	NM_009195	mouse
KCC2-147	TGCCCAGAAGTCTATCCCTAC	CACCAAGTTGCCATTCACAG	1347-1494	NM_029333	mouse
KCC2a-113	CCAGAGTCCCGCCGGCATTC	TCCGTGTCCGTGCTGTTG	61-172	XM_006499943	mouse
KCC2b-130	TCAACAACCTGACGGACTG	ATGTTCCTGCCATCGTACTC	5-134	NM_029333	mouse
KCC2a/b-108	CTGGACCAAGGATAAGTCAG	GGTTCAAGTTTTCCCACTCC	3018-3126	NM_029333	mouse
KCC3-113	GACAGAAACGTACCAGGAGAAG	GGTCCGGACGCATATTAAGTAG	2979-3078	NM_133648	mouse
KCC4-102	GACCTACTTCACCATGCTAGTT	ATAGACTTCTGGGCGTCTTTG	1251-1352	AF087436	mouse
L32-145	TTCGTTGCTGCTGCTTTCAC	ATGGTCTCTGGACGGCTAATG	28-173	NM_029271	mouse

Supplementary Figure 1: ***Predicted primary structure of β-cell mKCC2a, mKCC2a-S25 and mKCC2b proteins.*** **A.** Schema of the different KCC2 cDNAs cloned from MIN6 β-cells. Their coding regions are colored and the corresponding exons are indicated as grey arrowed boxes, whereas 5'- and 3'-UTRs are represented as filled lines. The relative positions of primer sets (opposite arrowheads) used for RT-PCR amplification of KCC2 mRNAs are also indicated. *GenBank* accession numbers KJ535320, KJ535321 and KJ535322 correspond to mKCC2a-S25, mKCC2a and mKCC2b, respectively. **B.** Quantitative PCR of KCC2a and KCC2b mRNAs expressed in MIN6 (*n*=5). Horizontal grey bars represent amplicons obtained with qPCR primers specific for KCC2a and KCC2b. Results are shown relative to total KCC2 mRNAs detected using primers that do not discern among KCC2 variants (KCC2-147, red horizontal bars). Expression levels of KCC2-S25 were inferred from qPCR data obtained with the primer set KCC2a/b-108 (black horizontal bars) where the antisense primer was specific to exon 25. **C.** *In silico* translation of KCC2 cDNAs. Underlined are the N-terminal residues unique to KCC2a or KCC2b. Shaded boxes depict predicted transmembrane domains. The splicing event in KCC2a-S25 is shown.

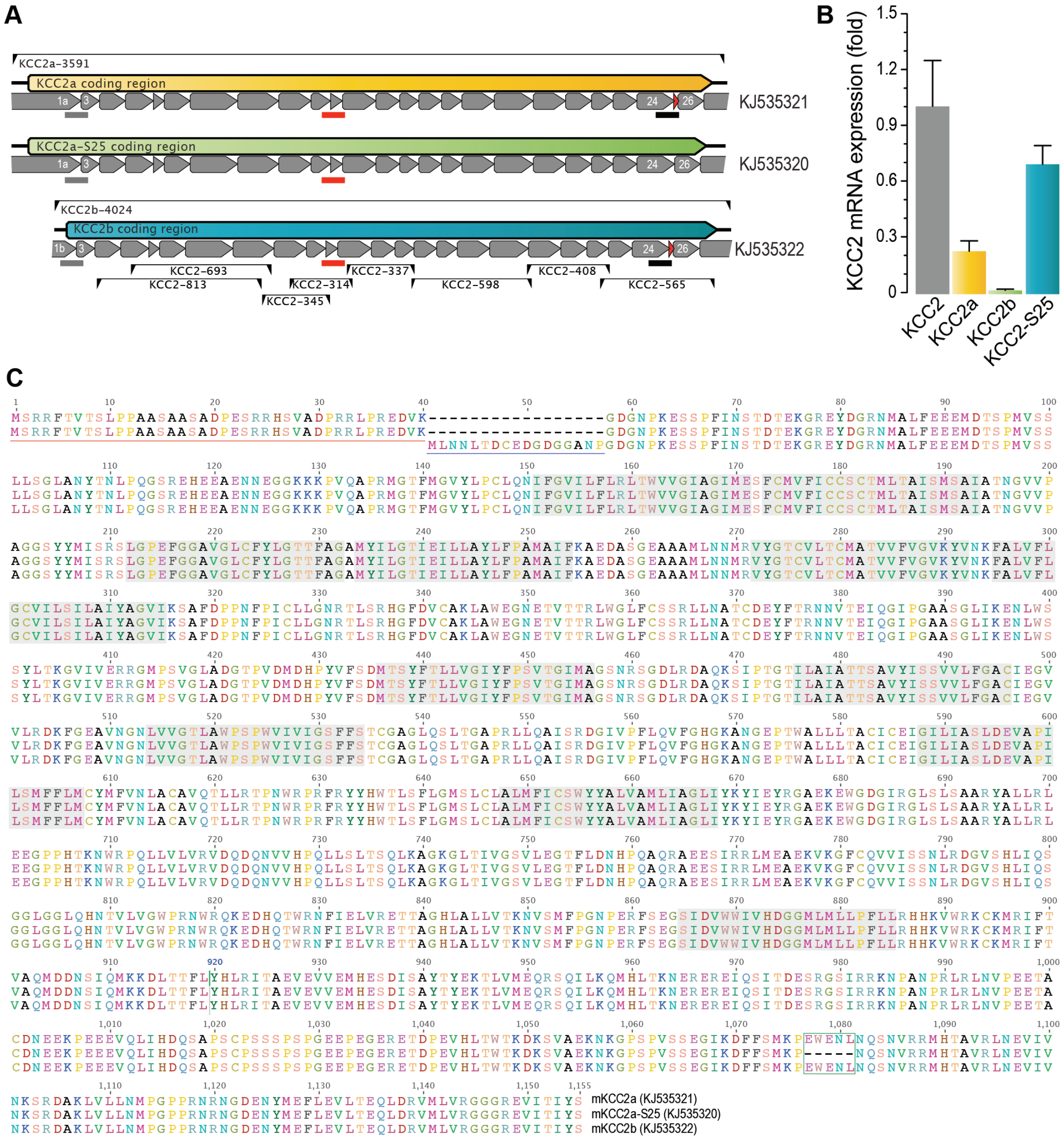

Supplementary Figure 2: ***Localisation of NKCC1, KCC2 and GLUT2 in the plasma membrane of islet cells.*** **A-C.** Mouse pancreatic sections were immunolabeled with antibodies recognising all cadherin members (pan-Cadherin) as plasma membrane marker and co-labeled against NKCC1 (A), KCC2 (B) or GLUT2 (C). Also shown in C, are insulin-labeled endocrine cells. **D-F.** Images obtained by using the Colocalization Finder tool of *ImageJ* and representing co-localisation (green) of pan-Cadherin and NKCC1 (D), KCC2 (E) and GLUT2 (F). Also shown in F, co-localization of pan-Cadherin and GLUT2 in in the plasma membrane of insulin producing β-cells. **G-i.** Shown are RGB profiles (signal intensities) obtained by using the *ImageJ* RGB Profiler tool along the yellow lines depicted in A, B and C. Green and red traces represent pan-Cadherin and NKCC1 (G), KCC2 (H) or GLUT2 (i) fluorescence intensities, respectively, whereas the blue trace represents that of insulin-labeled cells (i).

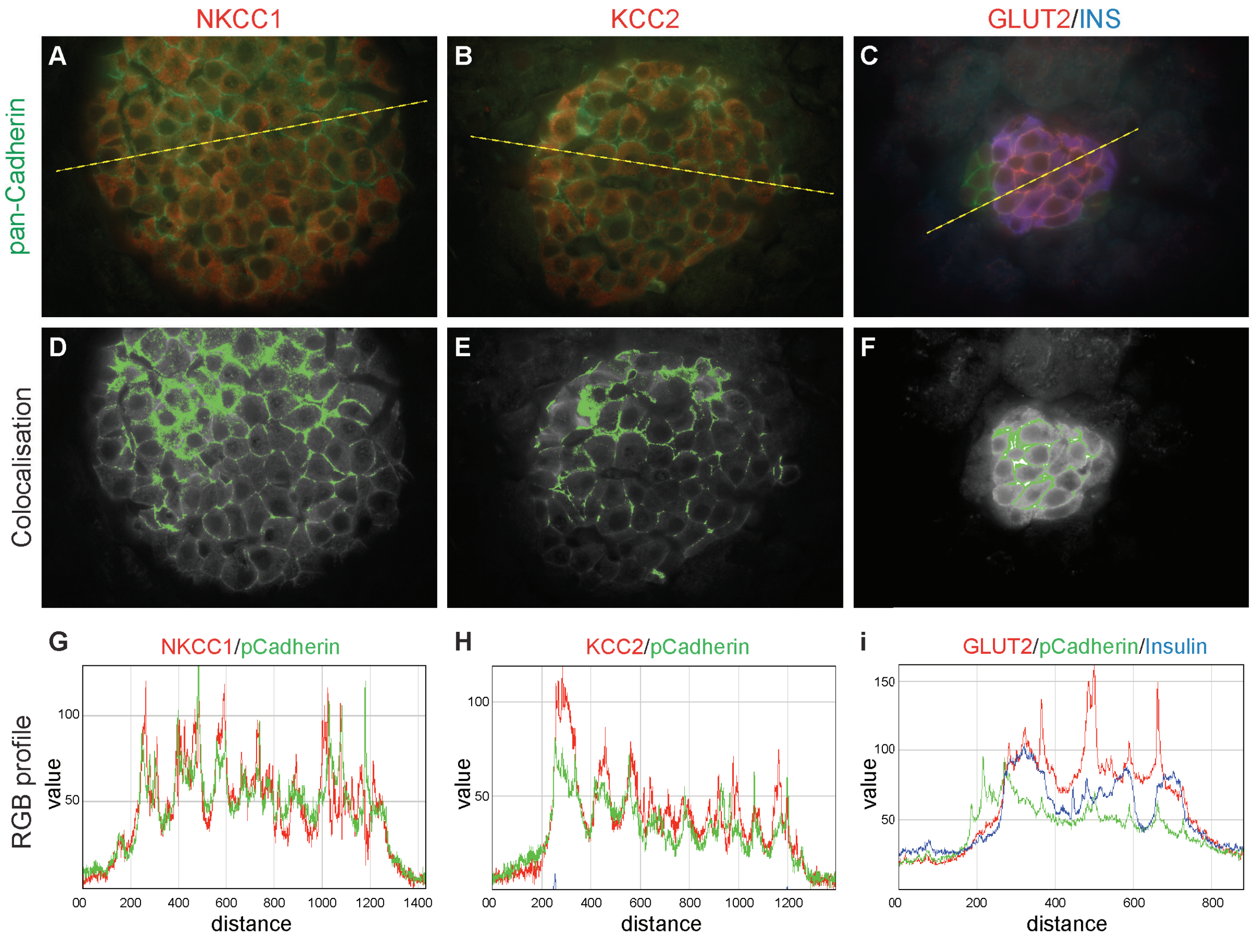

Supplementary Figure 3: ***KCC1, KCC3 and KCC4 mRNAs expression in MIN6 β-cells.* A.** Representative RT-PCR experiments demonstrating transcript expression of the main splice variants of KCC1, KCC3 and KCC4. Shown are amplicons of expected lengths for KCC1 (440bp, 529bp and 531bp), KCC3 (589bp), KCC3a (401bp), KCC3b (695bp), KCC3c (675bp), KCC3d (356bp) and KCC4 (663bp and 691bp). As a positive control, RT-PCR was directed by using GAPDH primers (555 bp). As a negative control of RT-PCR reactions, water was used instead of cDNA and performed with GAPDH primers. **B.** Quantitative PCR of KCC1, KCC2, KCC3 and KCC4 mRNAs. Results are expressed relative to KCC2 (total) transcripts. **C.** Schematic representation of KCC1, KCC3 and KCC4 transcripts of reference (*RefSeqs*) along with accession numbers. The coding regions and exons are indicated as black and grey arrowed boxes, respectively. The 5'- and 3'-untranslated regions (UTRs) are represented as filled lines and the relative positions of the primer sets used for transcript screening in RT-PCR experiments as opposite arrowheads. Amplicons quantified by qPCR primers are shown as colored horizontal bars.

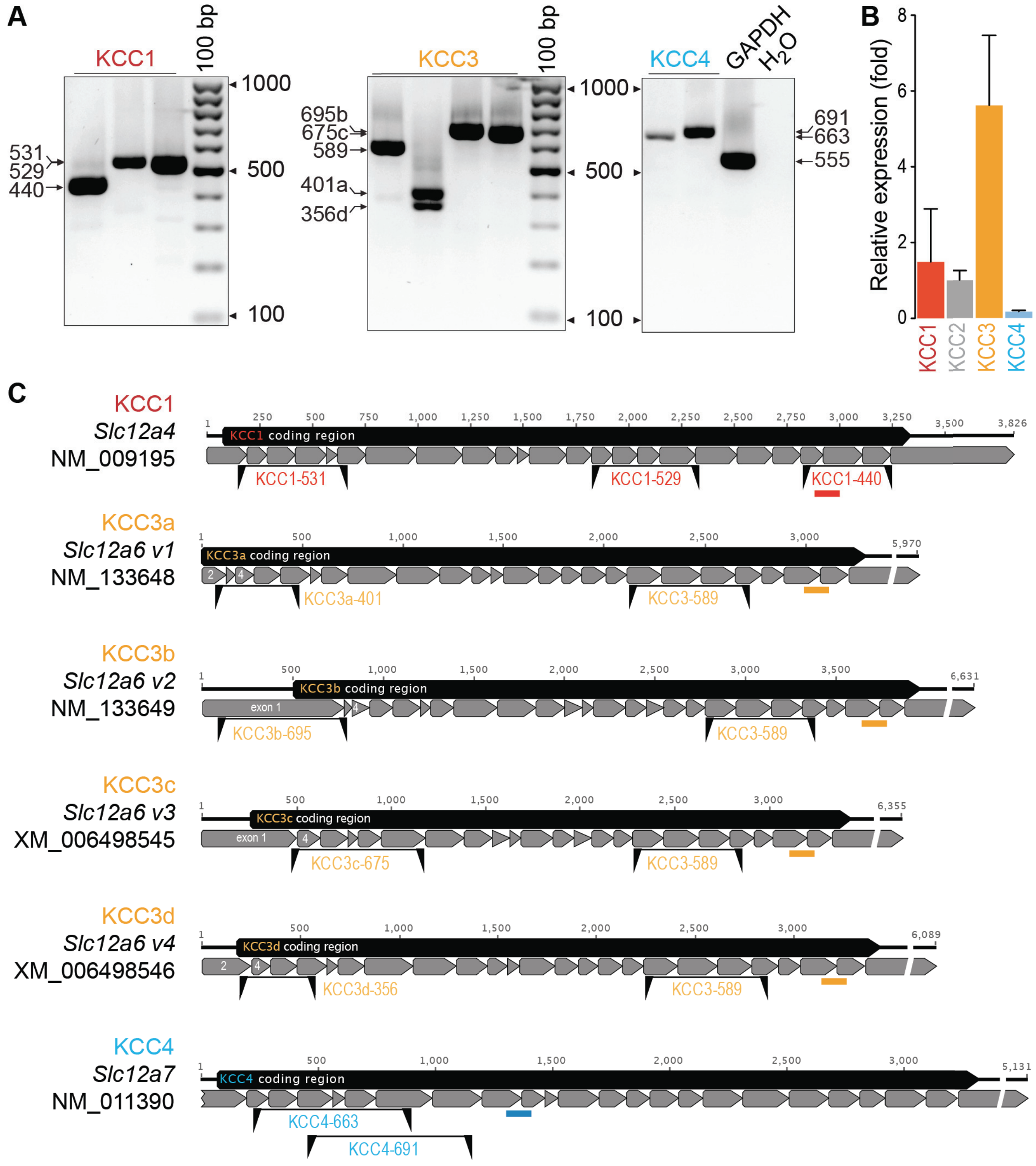

Supplementary Figure 4: ***Expression of mKCC2-S25 in medullary adrenal cells.*** **A-B.** Representative immunofluorescence microscopy images of the rat adrenal gland (20x, A or 100x, B) obtained using rabbit polyclonal KCC2 antibodies (07-432) and Cy3-conjugated secondary antibodies in 5 µm thick sections. KCC2 expression is shown in the medulla of the gland. Nuclei were counterstained with DAPI. Scale bars represent 50µm. **C.** Representative RT-PCR experiment showing KCC2 mRNA expression (KCC2a + KCC2b) detected by using the KCC2-565 primer set. GAPDH-555 primer set was used in the positive RT-PCR control reaction. As negative control, water instead of total cDNA was used in RT-PCR reactions with the KCC2-565 primer set. **D.** *MspI* digestion of KCC2-565 PCR products showing a restriction pattern consistent with the absence of exon 25 (362 bp). **E.** *MspI* digestion control of KCC2-565 products obtained using as PCR templates pCMV-*myc*.KCC2a and pCMV-*myc*.KCC2a-S25 cloning plasmids. **F-G.** Overexpression of *myc*-tagged mKCC2a-S25 in COS7 (F) and MIN6 β-cells (G) demonstrating that KCC2a-S25 cloned from MIN6 directs protein translation.

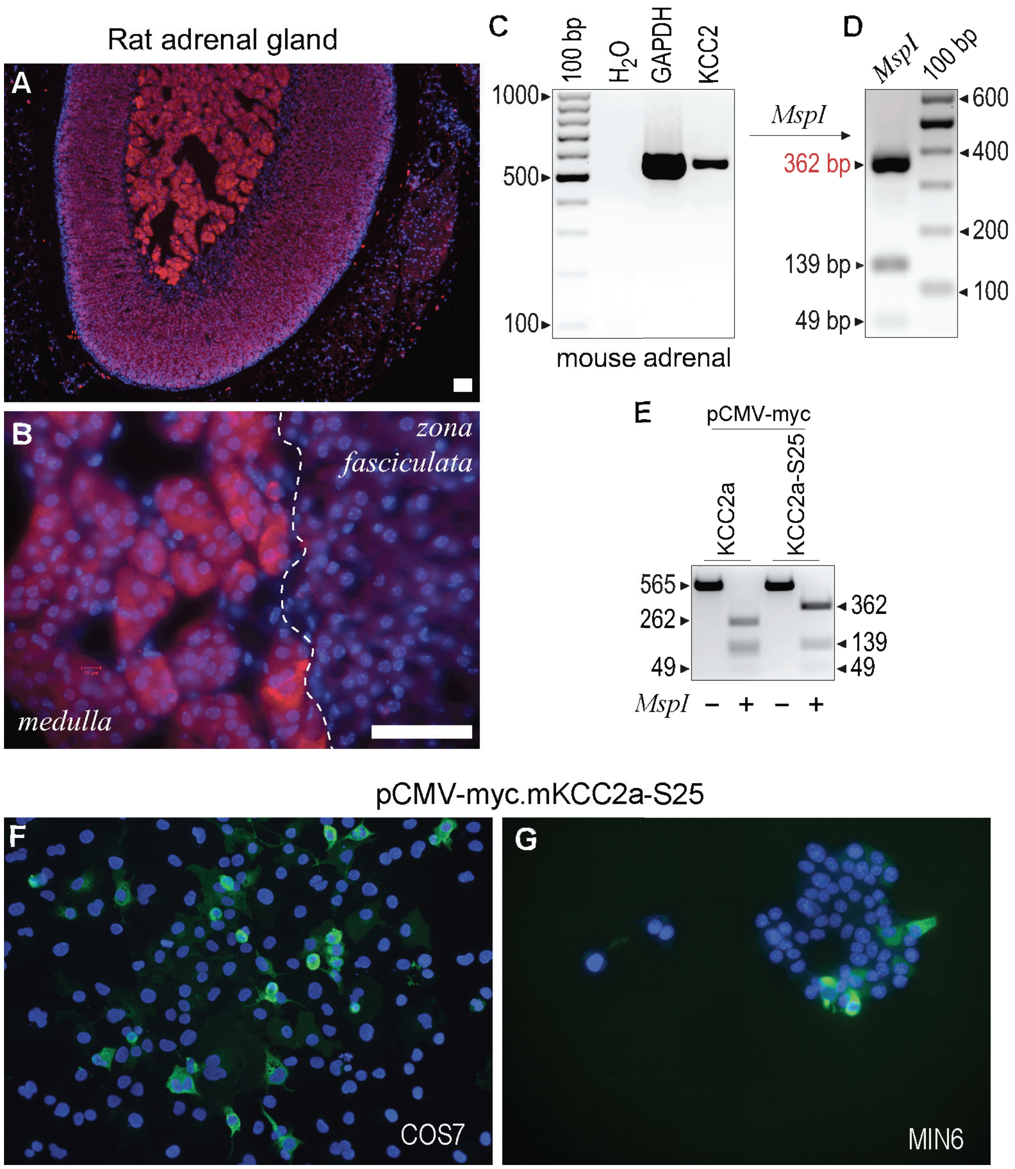

Supplementary Figure 5: ***KCC2 expression in glucagon-positive α-cells.*** **A-C.** Immunofluorescence microscopy images of pancreatic tissue obtained from an STZ-diabetic mouse immunolabeled against KCC2 (A, red), glucagon (B, green) and insulin (C, blue) using Cy3-, Alexa-fluor 488- or DyLight405-labeled secondary antibodies, respectively. **D-E.** Shown are superimposed images of A and C (D) and of A and B (E). **F.** Shown is a high magnification (100x) image of STZ pancreatic tissue to visualise and identify immunoreactive KCC2 in glucagon-positive and -negative cells of the islet (white arrows). **G-J.** KCC2 expression in αTC6 cells at the protein and mRNA levels. An original Western blot analysis of 100-50µg α-TC6 protein extracts developed with validated KCC2 antibodies (07-432) is shown (G) along with representative RT-PCR (H) and RT-qPCR using the KCC2-147 primer set which does not distinguish among KCC2 variants (see Table 2) (i) and a confocal immunofluorescence microscopy image of αTC6 cells attached to glass coverslips where white arrows indicate immunoreactive KCC2 near or at the plasma membrane region (J).

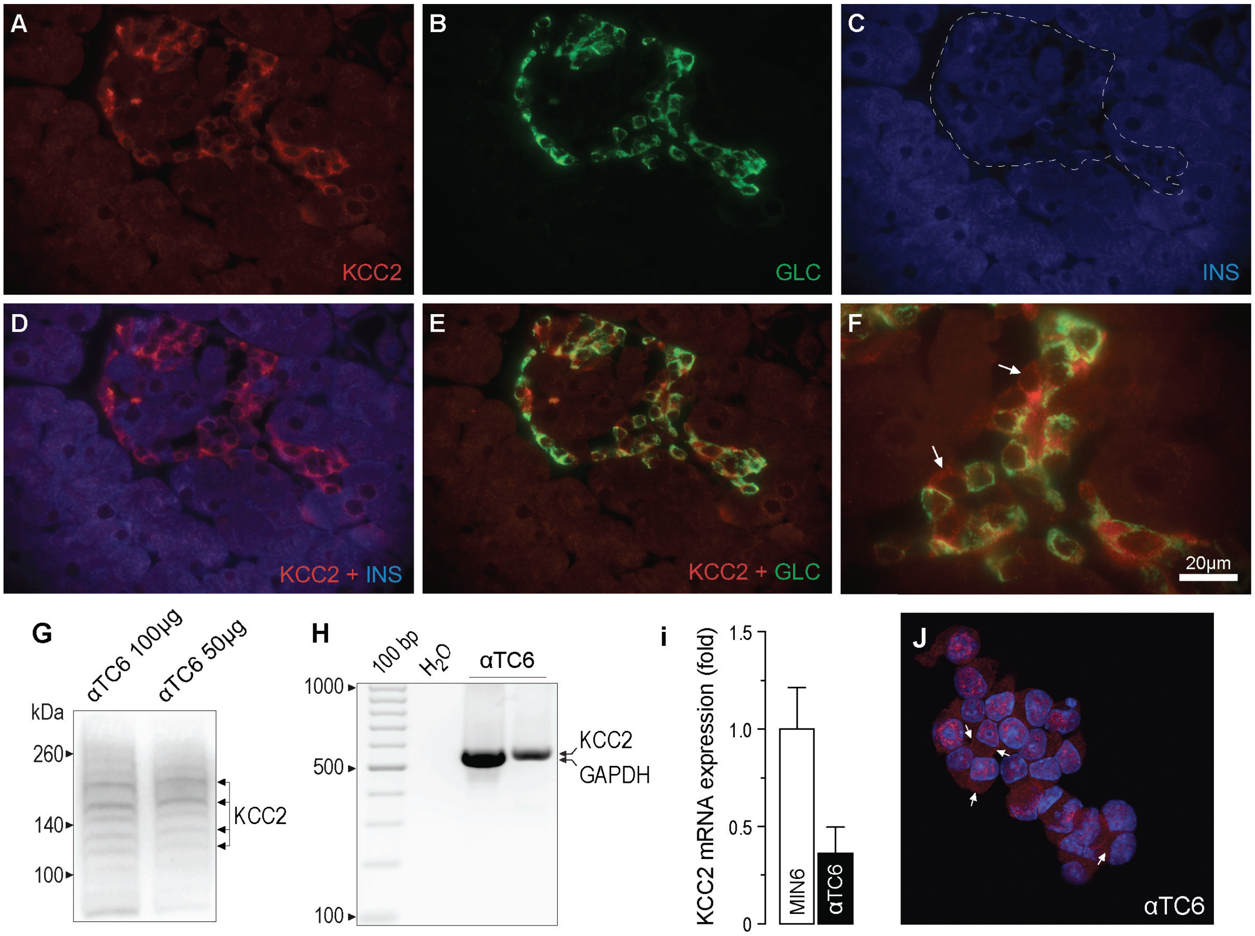

Supplementary Figure 6: ***Validation of KCC2 antibodies.*** **A-F.** Representative immunofluorescence microscopy images (20x) obtained using rabbit polyclonal KCC2 antibodies (07-432) and Cy3-conjugated secondary antibodies in 5 µm thick tissues sections positioned within the same chamber. KCC2 immunostaining was performed in mouse brain (A, hippocampus/white matter corpus callosum), spinal cord (B), kidneys (C), salivary glands (D), and mouse (E) and rat (F) pancreas. Nuclei were counterstained with DAPI. Scale bars represent 100 µm. **G-I**. Immunoblot analysis of the indicated protein extracts using 07-432 (G) and two monoclonal KCC2 antibodies: N1/12 (H) and N1/66 (I). Note that 07-432 detects KCC2 in mouse brain (50µg) as broad bands centered at ~150kDa but not in COS7 (100µg). N1/12 detects KCC2 in mouse brain as two bands (~125kDa and ~150kDa) and as a band of ~125kDa in mouse islets. N1/66 detects KCC2 in mouse brain as multiple bands, being the most prominent that of ~150kDa. In MIN6 and mouse islets, N1/66 detect KCC2 as bands of ~125-135kDa. **J-L.** Representative immunofluorescence microscopy images of COS7 (J) and MIN6 cells (K-L) immunostained with 07-432 (J), N1/12 (K) or N1/66 (L) antibodies coupled to Cy3-conjugated secondary antibodies. Nuclei were counterstained with DAPI. Scale bars represent 10µm. **M-O.** Representative immunofluorescence microscopy images of mouse pancreas slides labeled with 07-432 (M), N1/12 (N) or N1/66 (O) antibodies, coupled to Cy3-conjugated secondary ones. Nuclei in H-K were counterstained with DAPI, N-O were not counter-labeled. Scale bars represent 50µm.

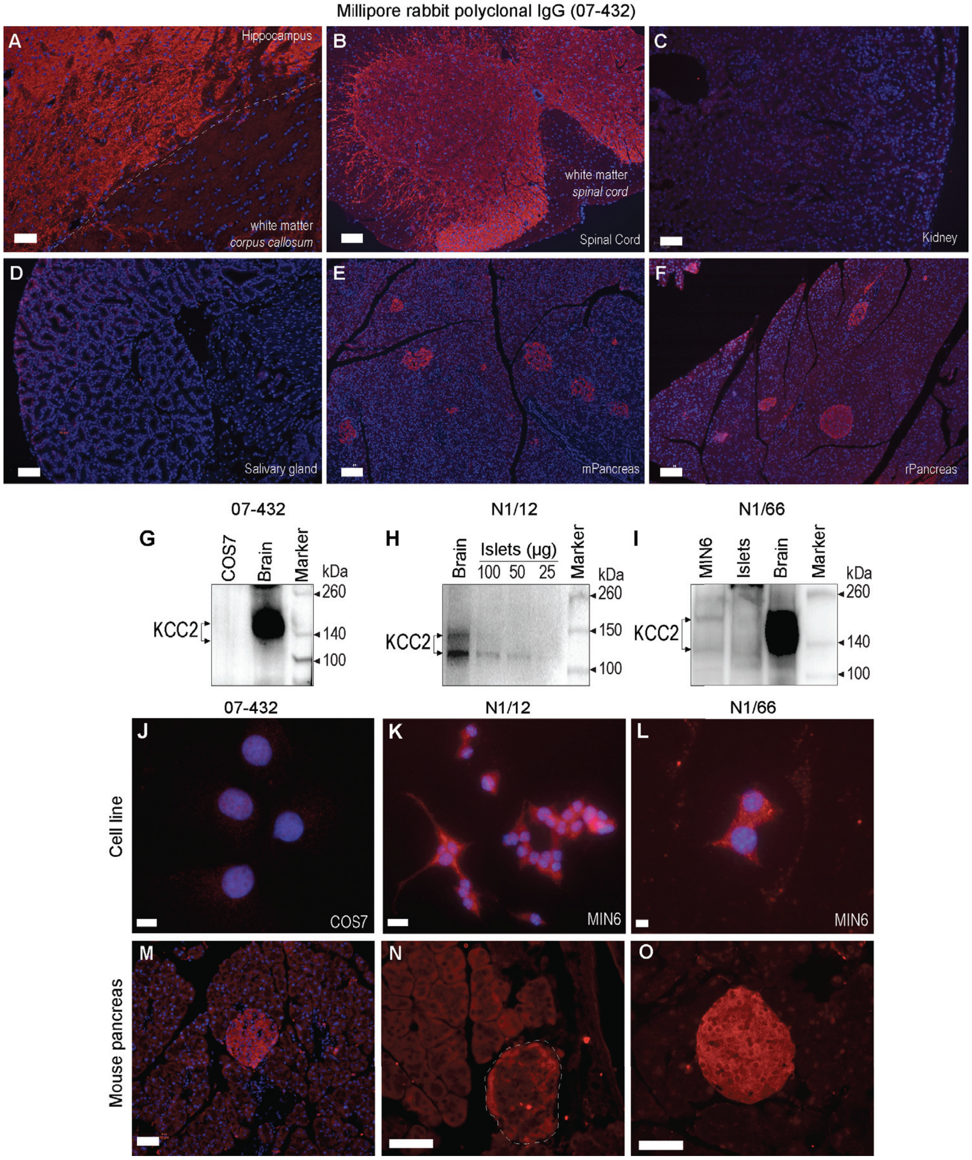

Supplementary Figure 7: ***Effects of VU0240511 and ML077 on the secretory response of MIN6 and the role of voltage-activated L-type Ca^{2+} channels*.** **A.** MIN6 β-cells were incubated in KRBH supplemented with 5.5mM glucose in the presence of 25μM KCC2 inhibitors: VU0240511 (filled bar) and ML077 (grey bar). Shown is a representative experiment performed in triplicate. Insulin released into the media and related to total cellular insulin content is expressed as percentage increase relative to Control (*p<0.05). **B.** Representative experiment of MIN6 β-cells incubated in KRBH plus 5.5mM or 12.5mM glucose in the presence of 25μM ML077 alone (filled bars) or in combination with 10μM nifedipine (Nif, grey bars). **C.** MIN6 β-cells were incubated in KRBH supplemented with 5.5mM glucose and challenged with 10μM GBC to evoke a rise in cytosolic Ca^{2+}. Subsequent addition of nifedipine (Nif, 5μM), an inhibitor of L-type Ca^{2+} channels, abolished GBC-evoked Ca^{2+} rises, as expected, and that in response to 25μM ML077 (see Figures 5B-C).

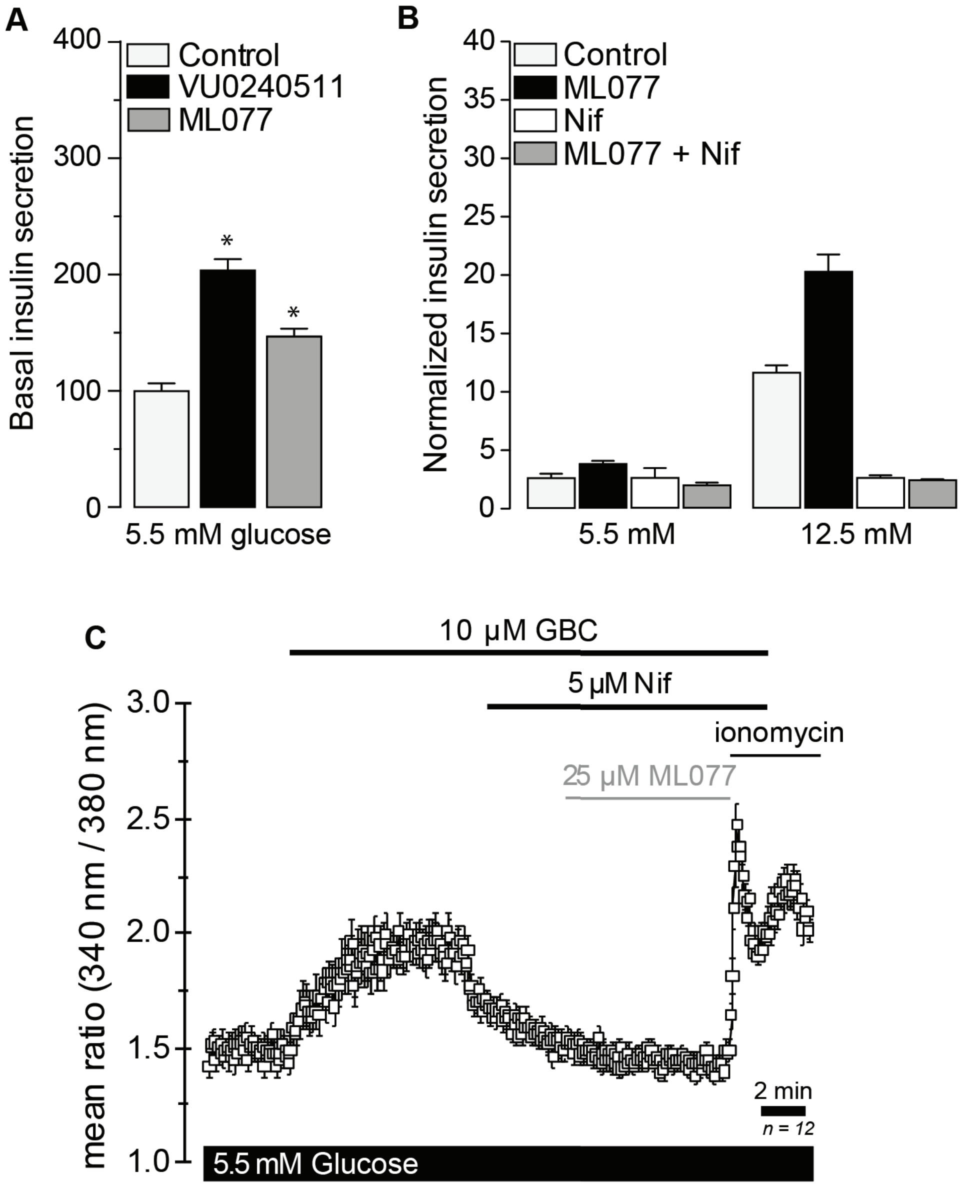

Original full-sized images

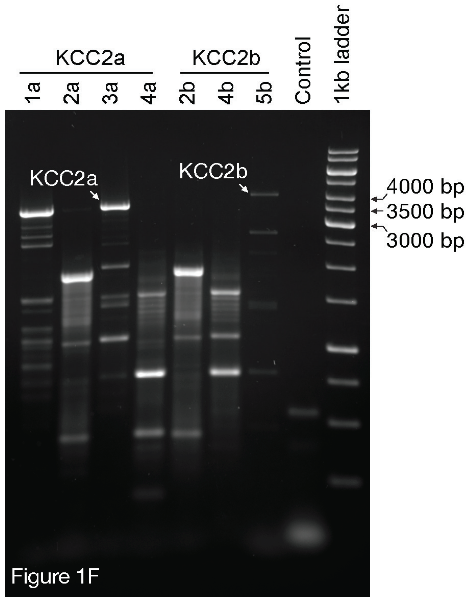

Figure 1F

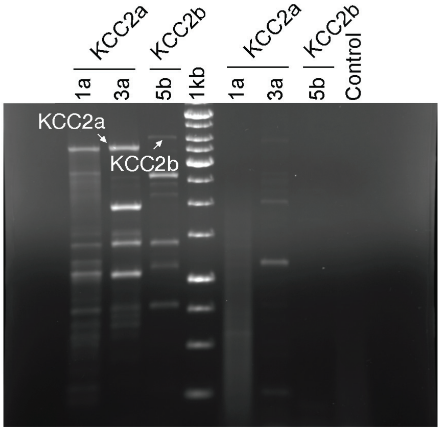

1a (mKCC2a-1s + mKCC2-1a)=3543 bp
2a (mKCC2a-1s + mKCC2-2a)=3568 bp
3a (mKCC2a-1s + mKCC2-3a)=3591 bp
4a (mKCC2a-1s + mKCC2-4a)=3713 bp
2b (mKCC2b-1s + mKCC2-2a)=3725 bp
4b (mKCC2b-1s + mKCC2-4a)=3869 bp
5b (mKCC2b-1s + mKCC2-5a)=4024 bp

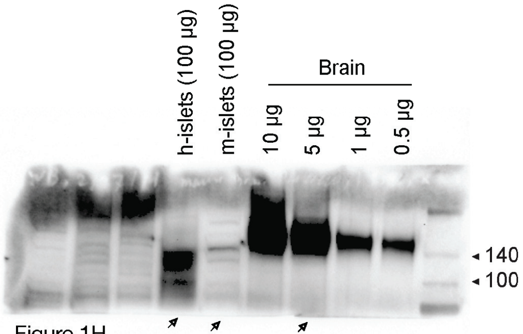

Figure 1H

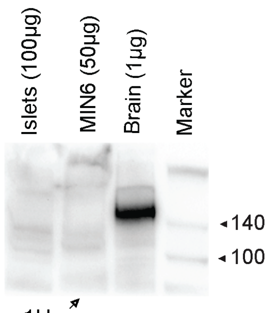

Figure 1H

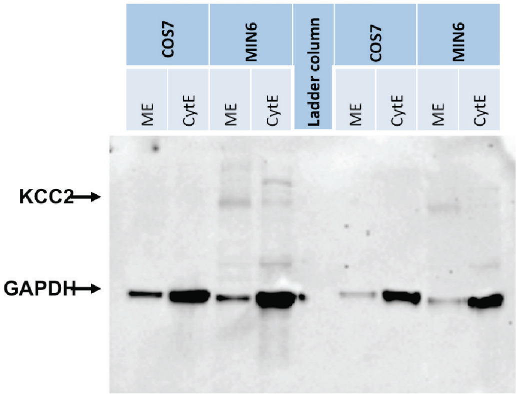

Figure 1H
